# Supplementary material for: Dehydration regulates structural reorganization of dynamic hydrogels
Source: Nat Commun. 2024 Aug 12;15:6886. doi: 10.1038/s41467-024-51219-7 (PMC11317490; doi:10.1038/s41467-024-51219-7)
Supplement: Supplementary file 1 — Supplementary Information [file 41467_2024_51219_MOESM1_ESM.pdf]

Supplementary Information

Dehydration regulates structural reorganization of dynamic hydrogels

Dan Xu[1], Xintong Meng[1], Siyuan Liu[1], Jade Poisson[1], Philipp Vana[2] and Kai Zhang[1,3 *]

Address

[1] Sustainable Materials and Chemistry, Department of Wood Technology and Wood-based Composites, University of Göttingen, Büsgenweg 4, D-37077 Göttingen, Germany.

[2] Institute of Physical Chemistry, University of Göttingen, Tammannstr. 6, D-37077 Göttingen, Germany.

[3] Biotechnology Center (Biotechnikum), University of Göttingen, Büsgenweg 2, D-37077 Göttingen, Germany.

[*]Corresponding author. E-mail: kai.zhang@uni-goettingen.de

This file includes:

Supplementary Figure 1-48

Supplementary Notes 1-5

Supplementary References

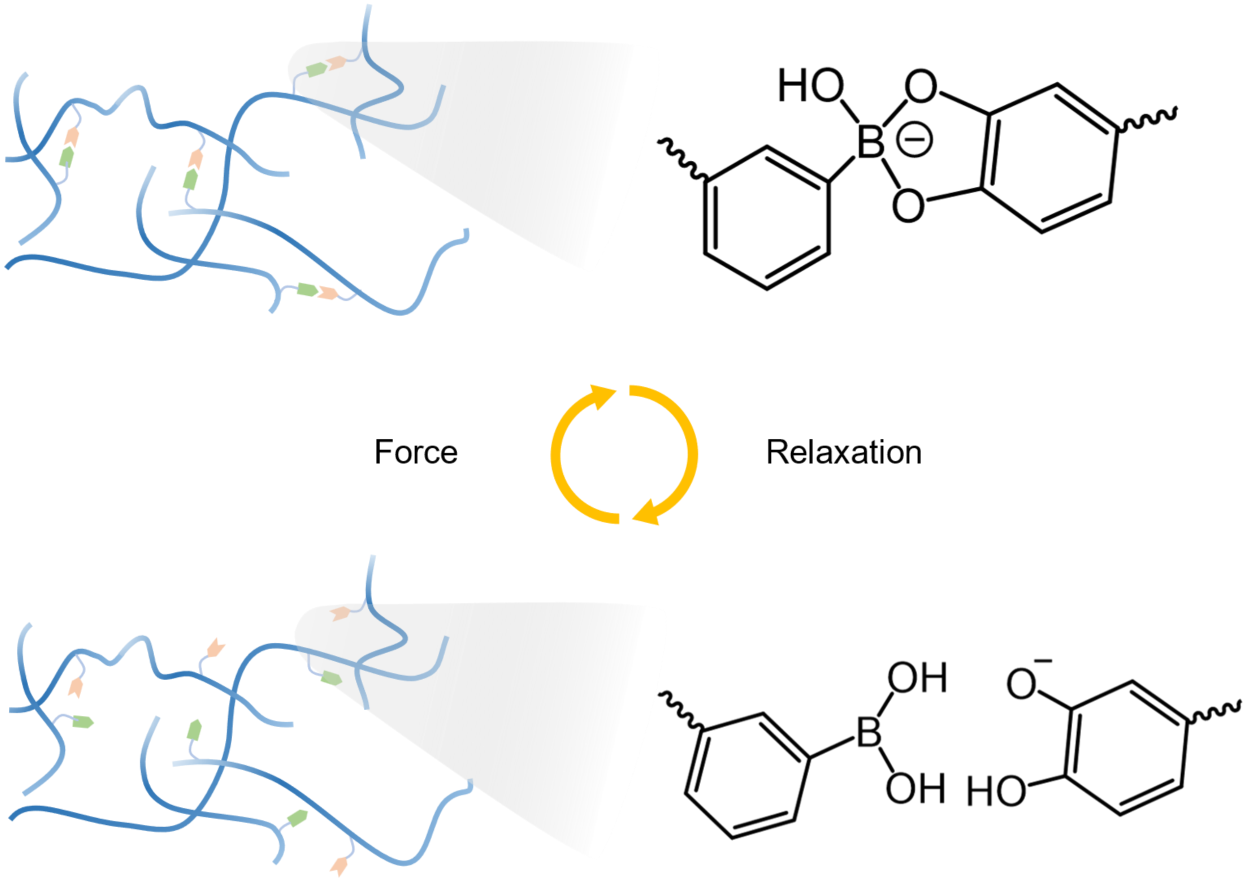

Supplementary Figure 1. A dynamic reorganization in dynamic hydrogels based on boronate ester with external force. Boronate esters are characterized by tunable mechanical properties and a thorough investigation of their properties[1, 2, 3].

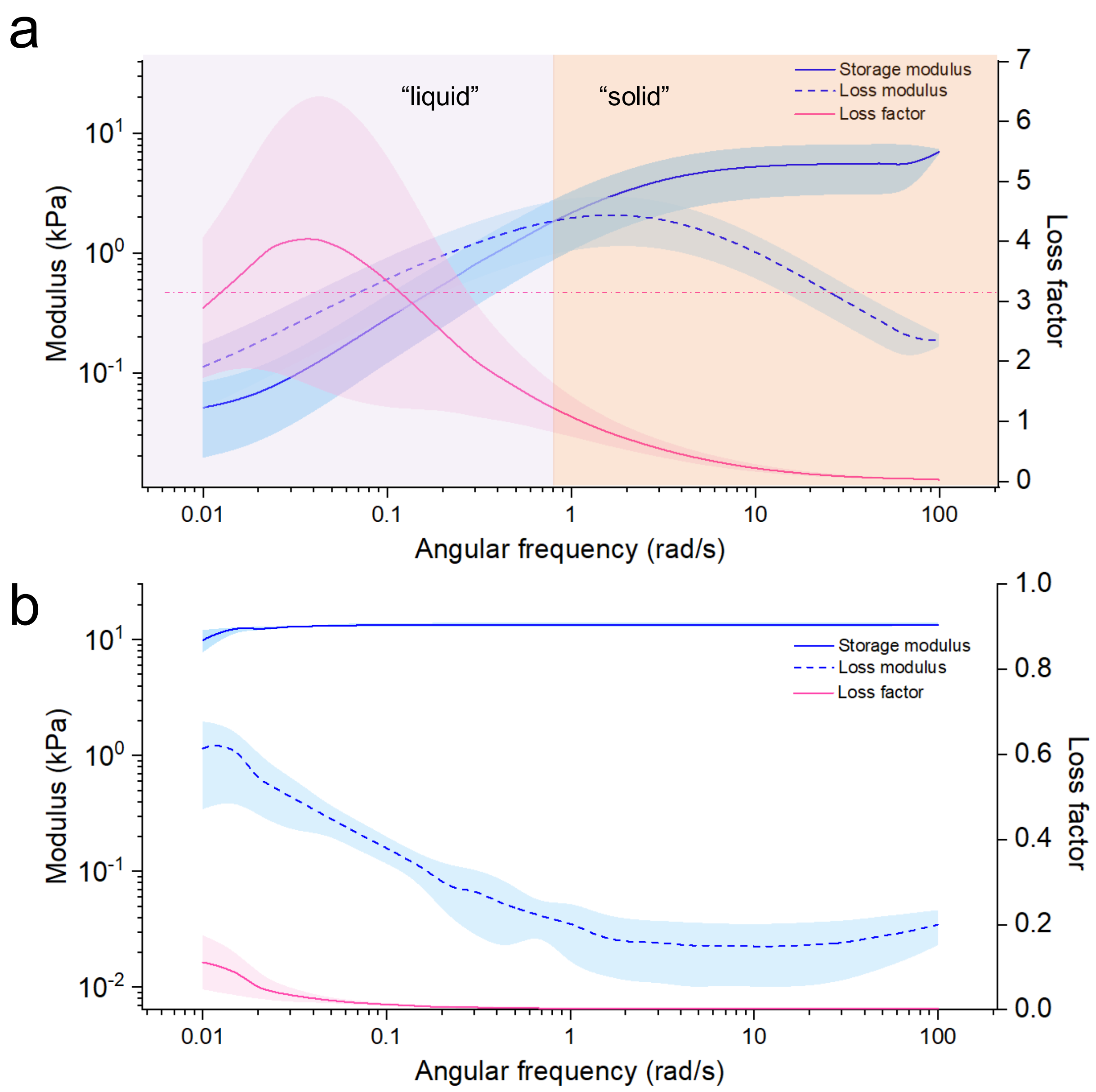

Supplementary Figure 2. Frequency sweep curves of hydrogels. a). Dynamic hydrogels. b). Covalent hydrogel. Individual experiments N ≥ 4.

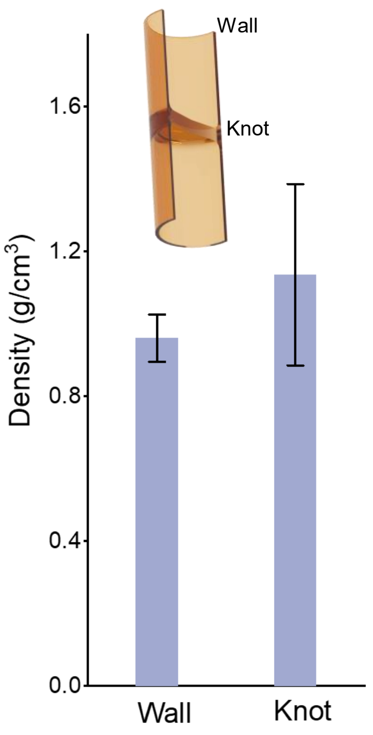

Supplementary Figure 3. The density of xerogel walls and knots in the resulting hollow xerogel tubes. N = 5 individual experiments. Error bars are SD.

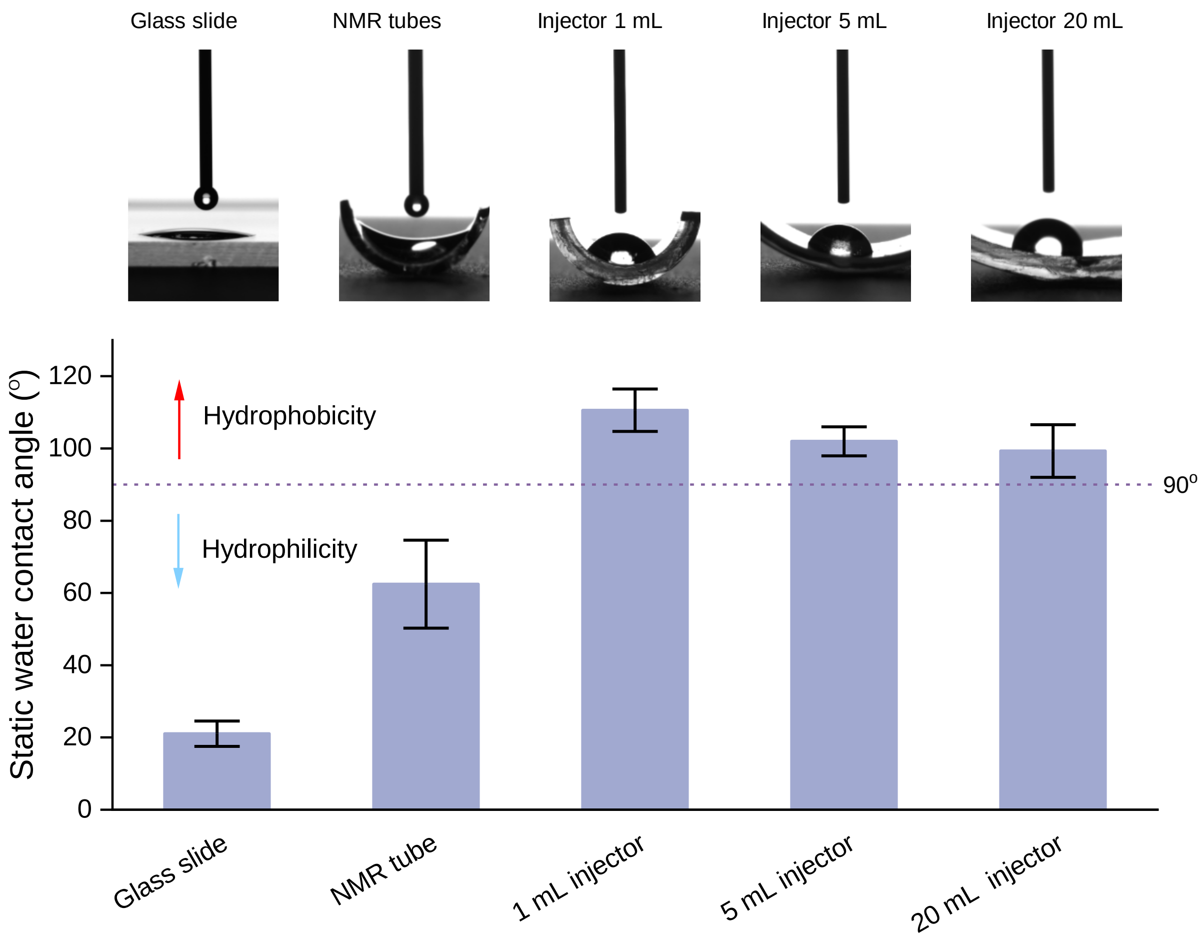

Supplementary Figure 4. The static water contact angles of various substrates. The NMR tubes are manufactured from glass. The injector wall is constructed from polypropylene. As the diameters of different injectors are different, the values of static water contact angles only reflect the apparent contact angle. N = 5 individual experiments. Error bars are SD.

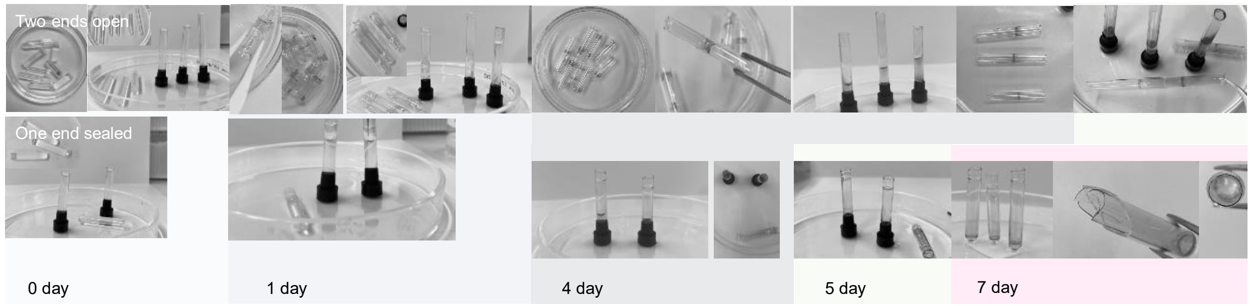

Supplementary Figure 5. Dehydration of dynamic hydrogels in tubes with hydrophilic inner surface. The hollow tubes with knots were also formed.

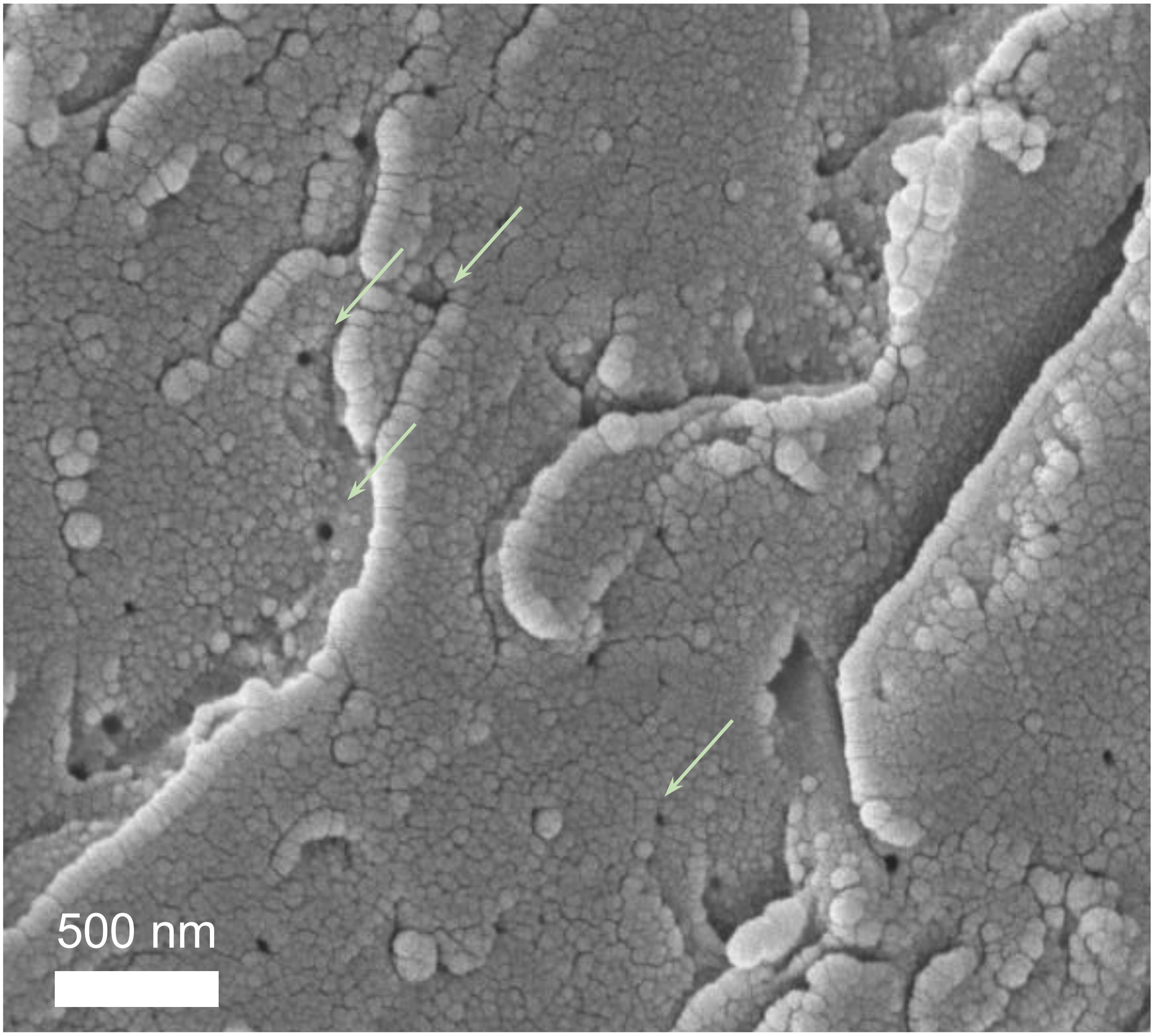

Supplementary Figure 6. A typical porous microstructure of xerogel hollow tubes around all areas of xerogel hollow tubes.

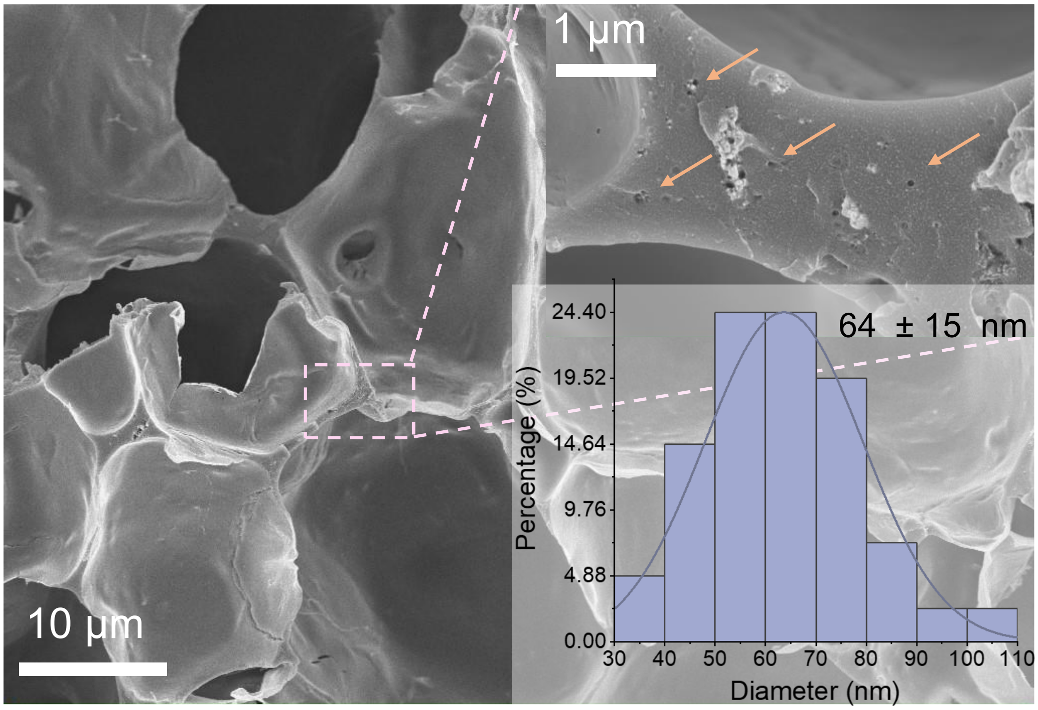

Supplementary Figure 7. The pores were located in surface regions between the apertures of the lyophilized hydrogel. One potential explanation for this occurrence is the creation of ice crystals during the process of freezing[4].

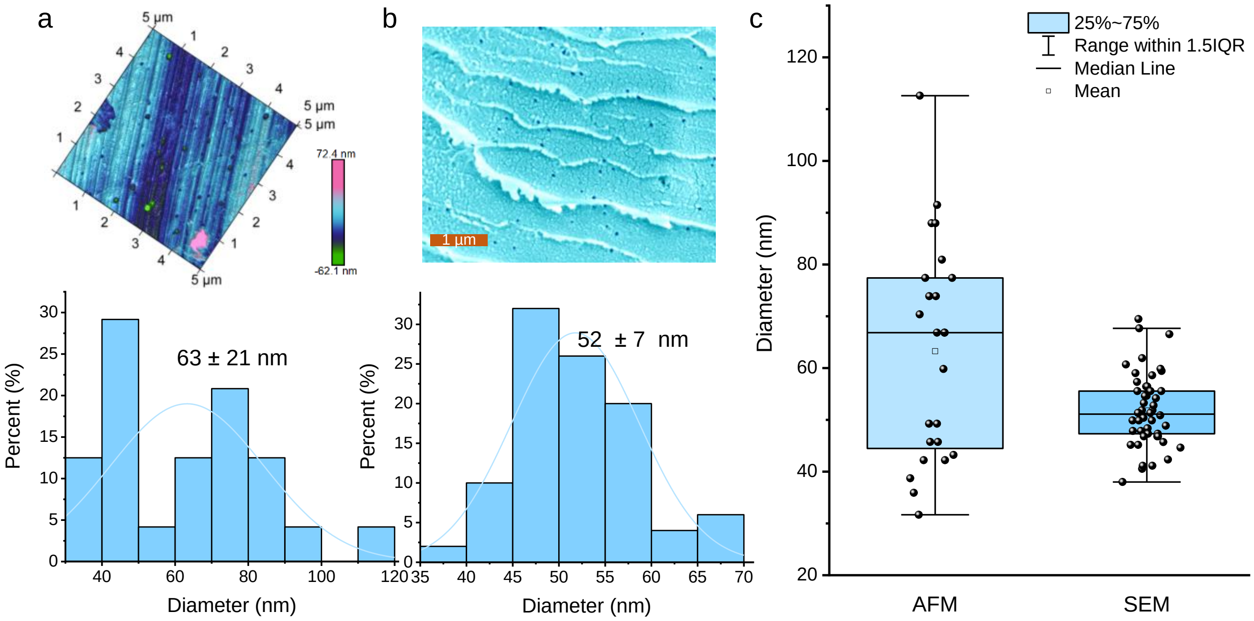

Supplementary Figure 8. A universal porous structure in hollow xerogel tubes. a). The 3D AFM image and statistical analysis of pore size based on AFM images. b). The SEM image (false color) and statistical analysis of pore size based on SEM images. c). The statistical analysis of pore sizes.

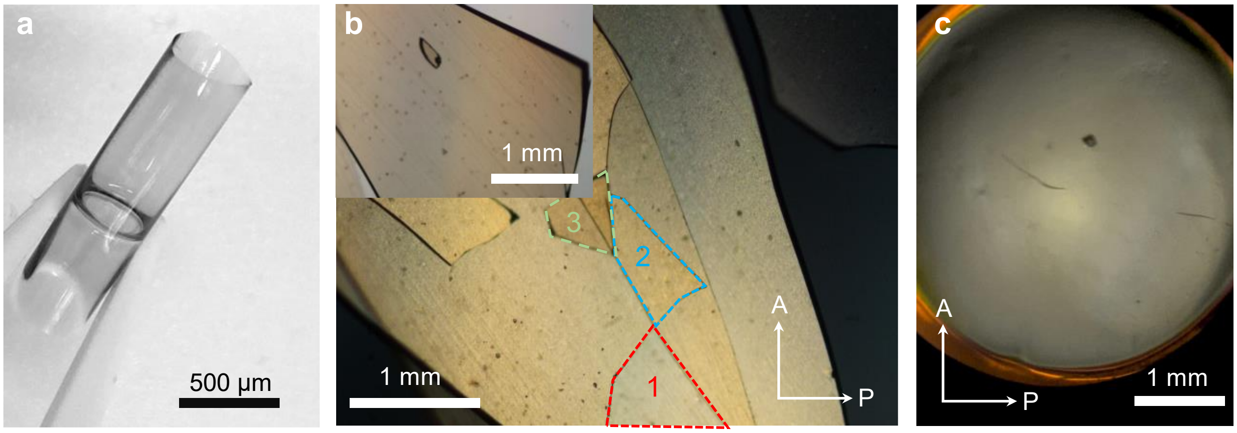

Supplementary Figure 9. The images of hollow xerogel tubes. a). The snapshot of hollow xerogel tube. b). Polarized optical microscopy (POM) image of xerogel wall. The numbers refer to the stacking layers of the xerogel wall. Inset: OM images of xerogel wall. c). POM image of xerogel knot.

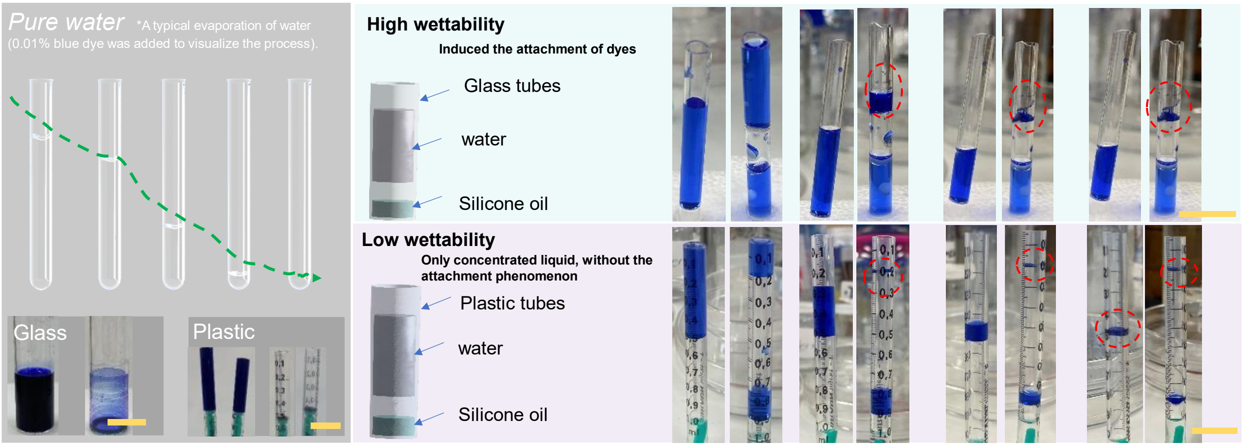

Supplementary Figure 10. A typical CCR process of water evaporation in tubes. The resulting patterns also vary depending on the hydrophilicity of the substrate. Scale bars are 1 cm.

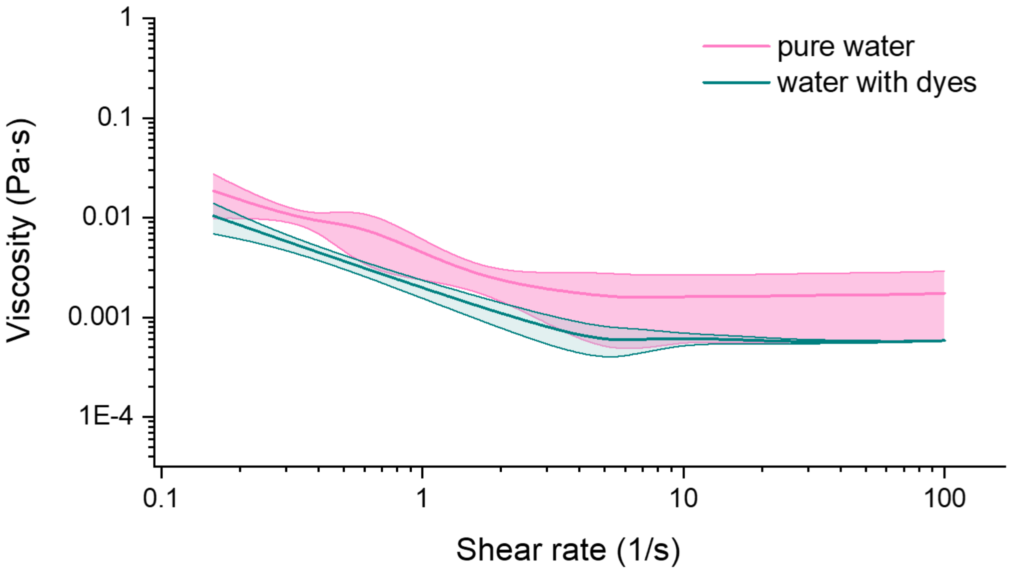

Supplementary Figure 11. Viscosity of pure water or dyeing water. When small amounts of dyes (less than 0.01 wt%) were added to make the drying process visible, the flow properties of the water remained almost the same.

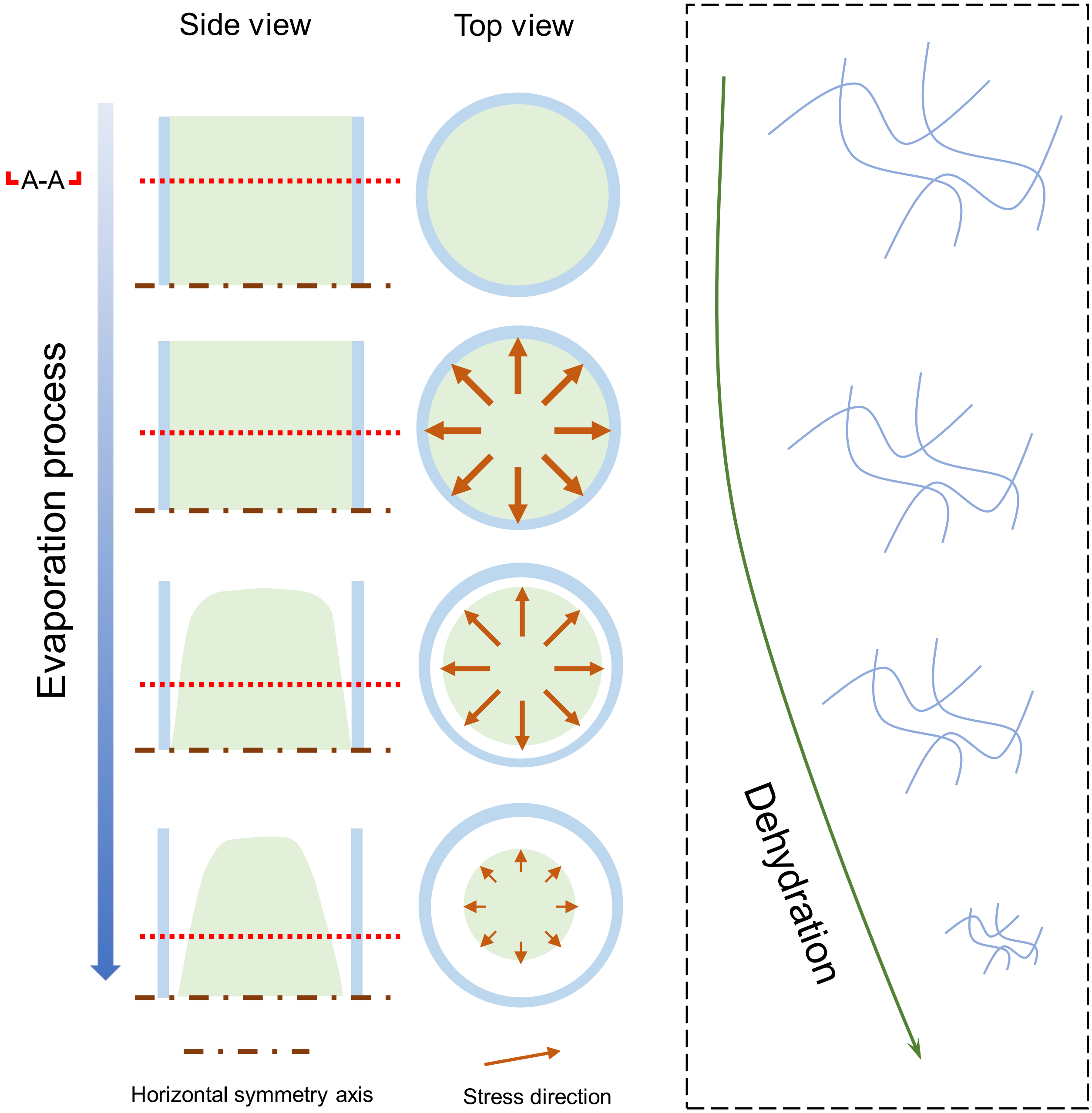

Supplementary Figure 12. The isotropic shrinkage of covalent hydrogel in dehydration process. As for the hydrogels that was covalently crosslinked or materials possessing few dynamic bonds, the internal stress was enhanced due to evaporation process. In porous materials, water can be stored in small capillaries or pores. The loss of water through evaporation reduces the fluid pressure within these capillaries, resulting in the contraction of the hydrogels. Thus, the loss of water volume and resulting capillaries action causes hydrogels to shrink and stabilize the observed morphologies.

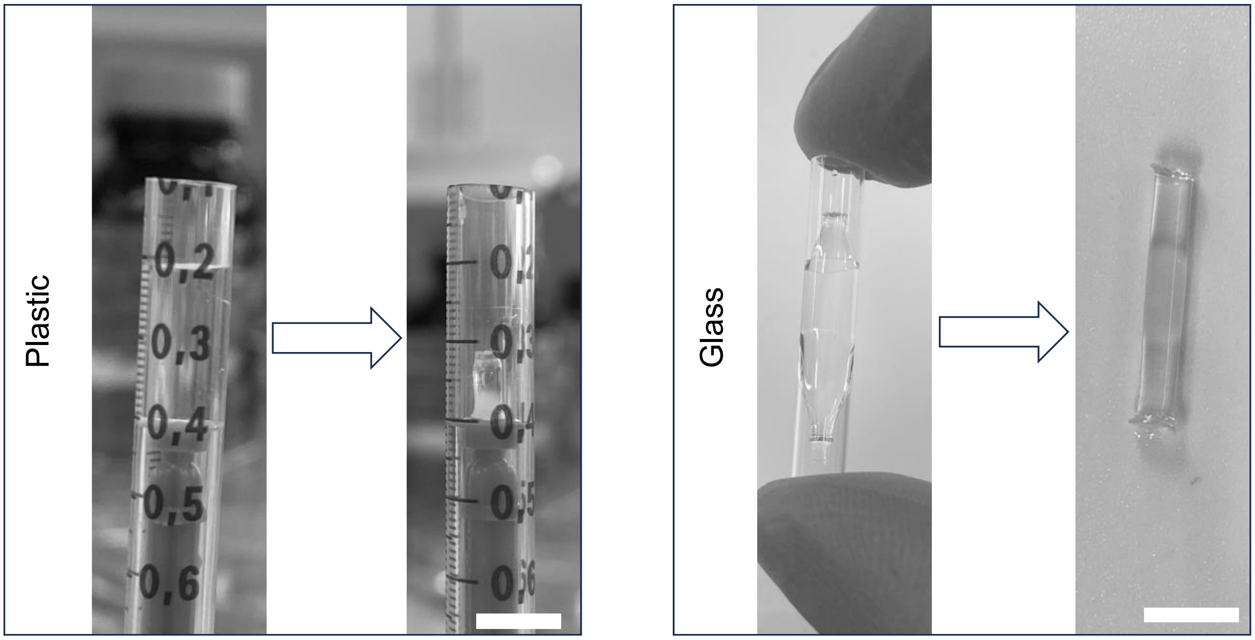

Supplementary Figure 13. The dehydration of covalently crosslinked hydrogel in plastic tube or glass tube. Scale bars are 0.5 cm.

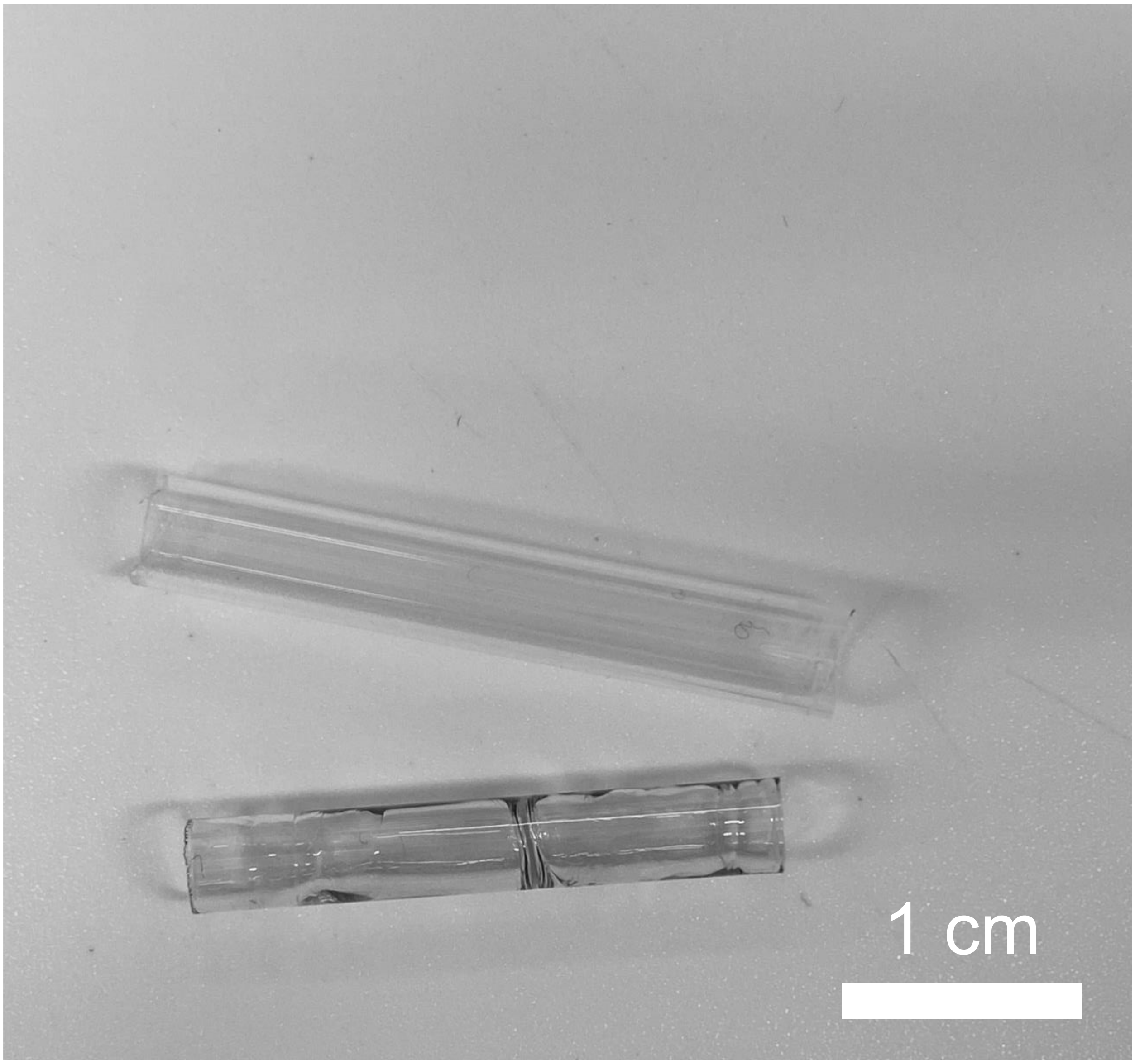

Supplementary Figure 14. The resulting xerogel tubes of dynamic hydrogels (monomer concentration 4 M) in glass tubes.

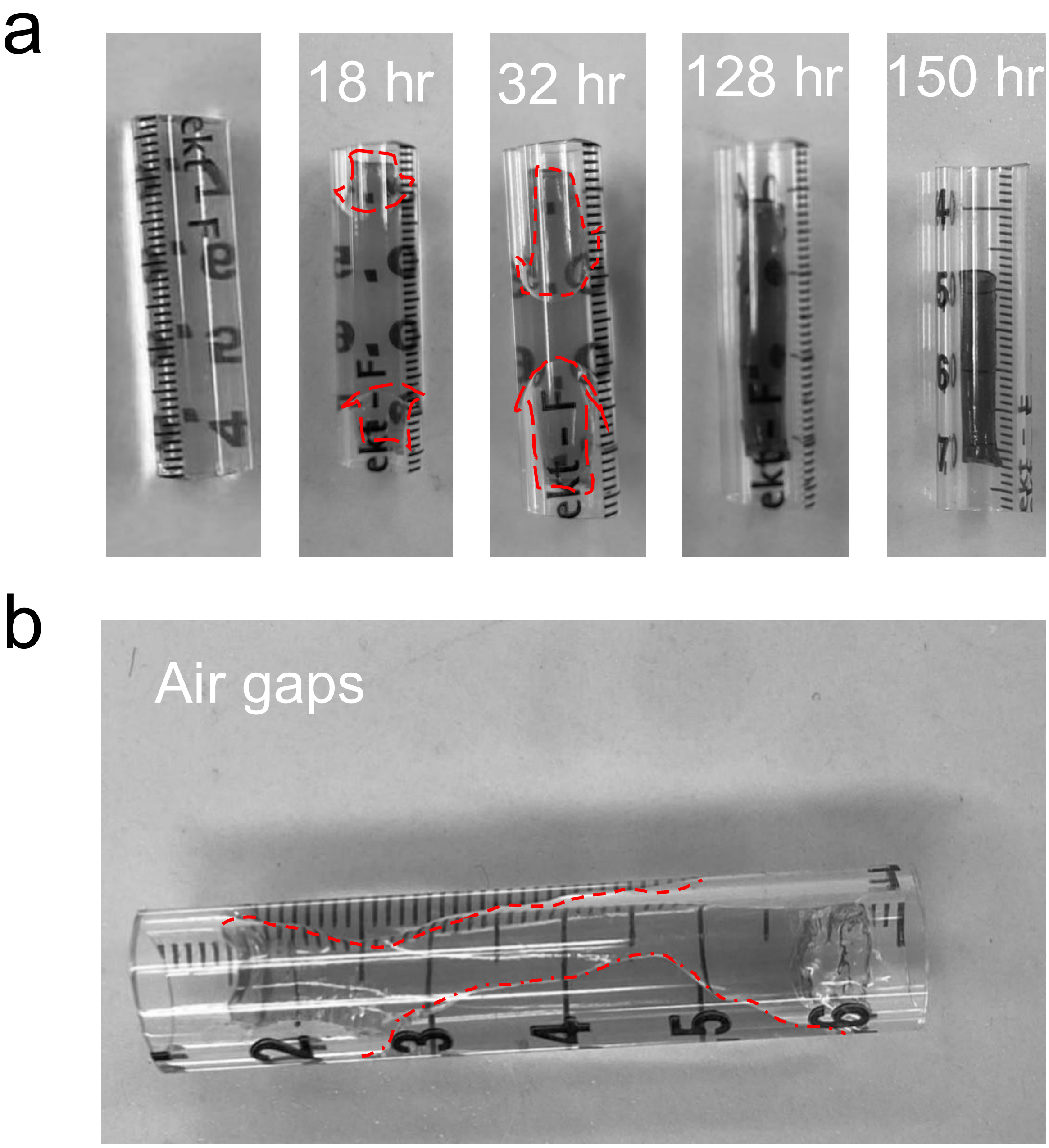

Supplementary Figure 15. The failure of dynamic hydrogel-wall interface during dehydration due the system's sensitivity to initial characteristics. a). The hydrogel-wall interface failed in the early stages of dehydration because of the small gaps present around the interface. These gaps can be caused either by defects around the wall or during the trimming of the outer tubes. b). The voids surrounding the hydrogel wall result from the buildup of minuscule gaps.

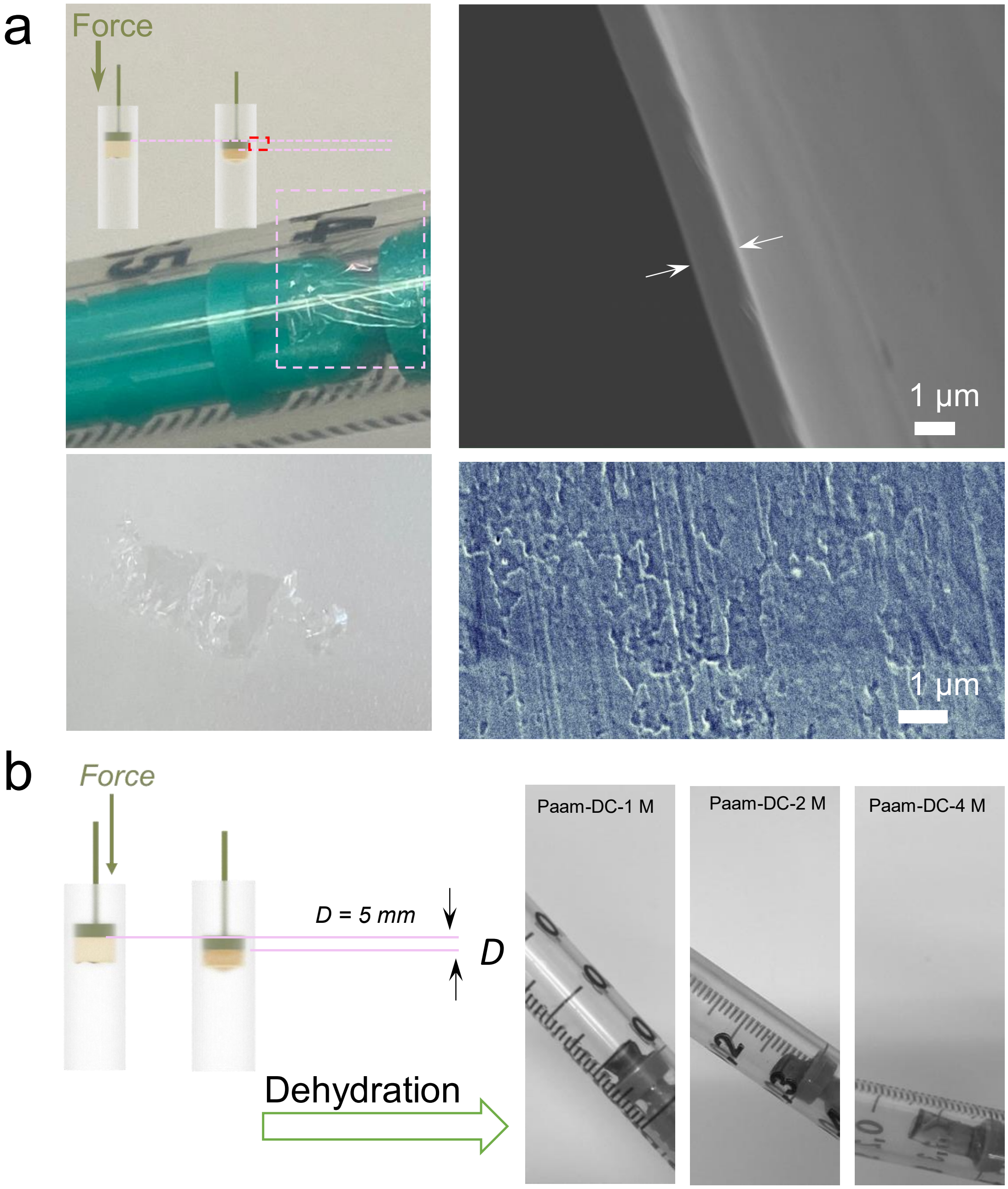

Supplementary Figure 16. The destruction of the unblemished hydrogel-wall interface induced isotropic shrinkage of dynamic hydrogel during dehydration. a). The residual film was found after the compression (0.1 mm/min), demonstrating the presence of a tough anchored layer in hydrogel-wall interface. The thickness of the layer was about 1 μm. The trenches were found on these anchored layers. Note that the color SEM image is false color. b). The unblemished hydrogel-wall interface was destroyed via external shear stress (compression ratio 100%). Then, with the newly generated hydrogel-wall interface the isotropic shrinkage of dynamic hydrogels was found. The eroded conformality led to a weaker interaction between the hydrogel and the wall interface, thus suggesting it as the underlying cause.

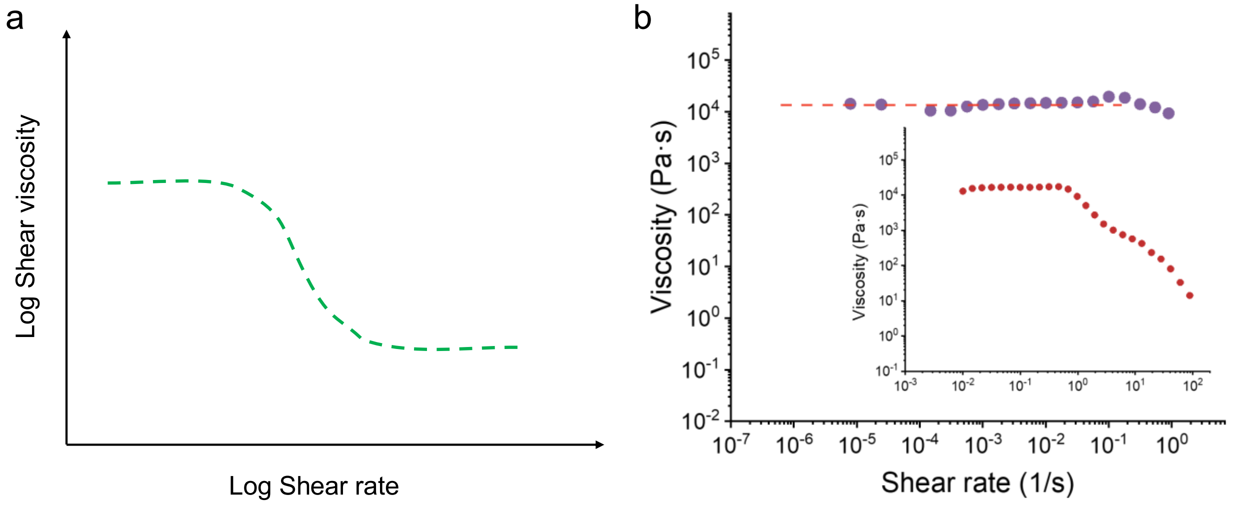

Supplementary Figure 17. An extrapolation method for assessing the viscosity of hydrogels. a). Typical flow curves for shear thinning fluids with a zero-shear viscosity. b). The viscosity of hydrogels at ultra low shear rates. The plateau is evident.

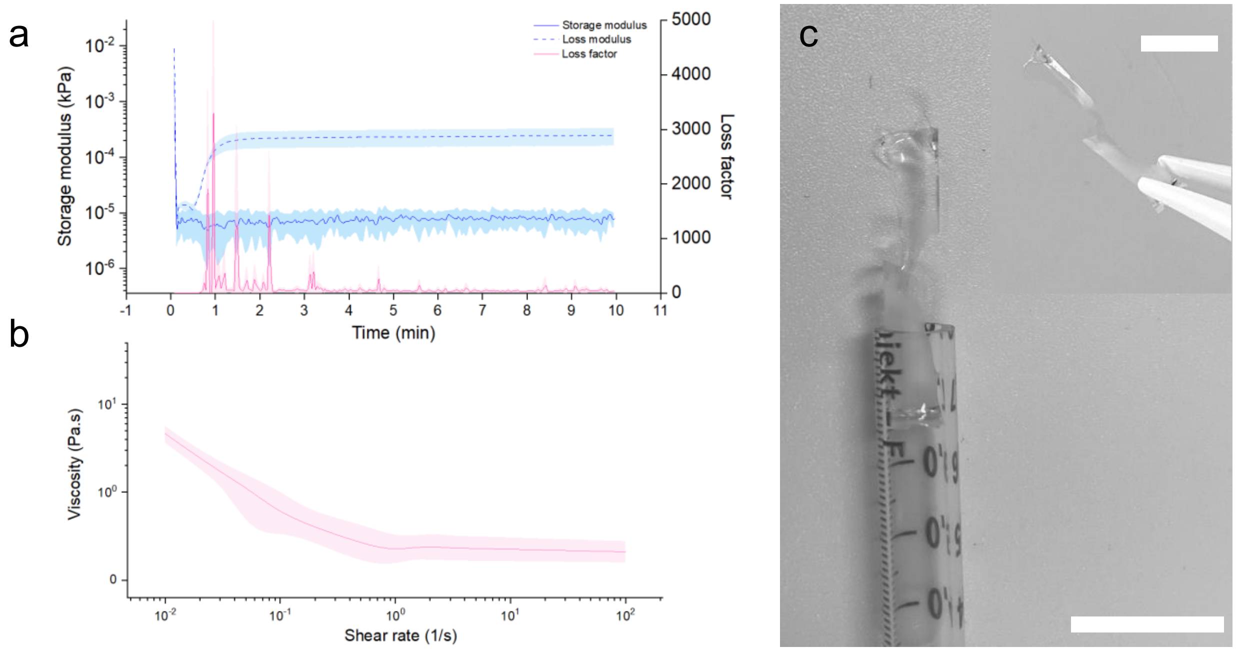

Supplementary Figure 18. The rheological properties of non-crosslinked hydrogels. a). The evolution of rheological properties of precursor of non-crosslinker hydrogel. b). The viscosity of non-crosslinked hydrogel. c). The snapshot of the resulting xerogel after the dehydration of non-crosslinked hydrogels. Inset: the xerogel upon removal from the tube. Scale bars are 1 cm.

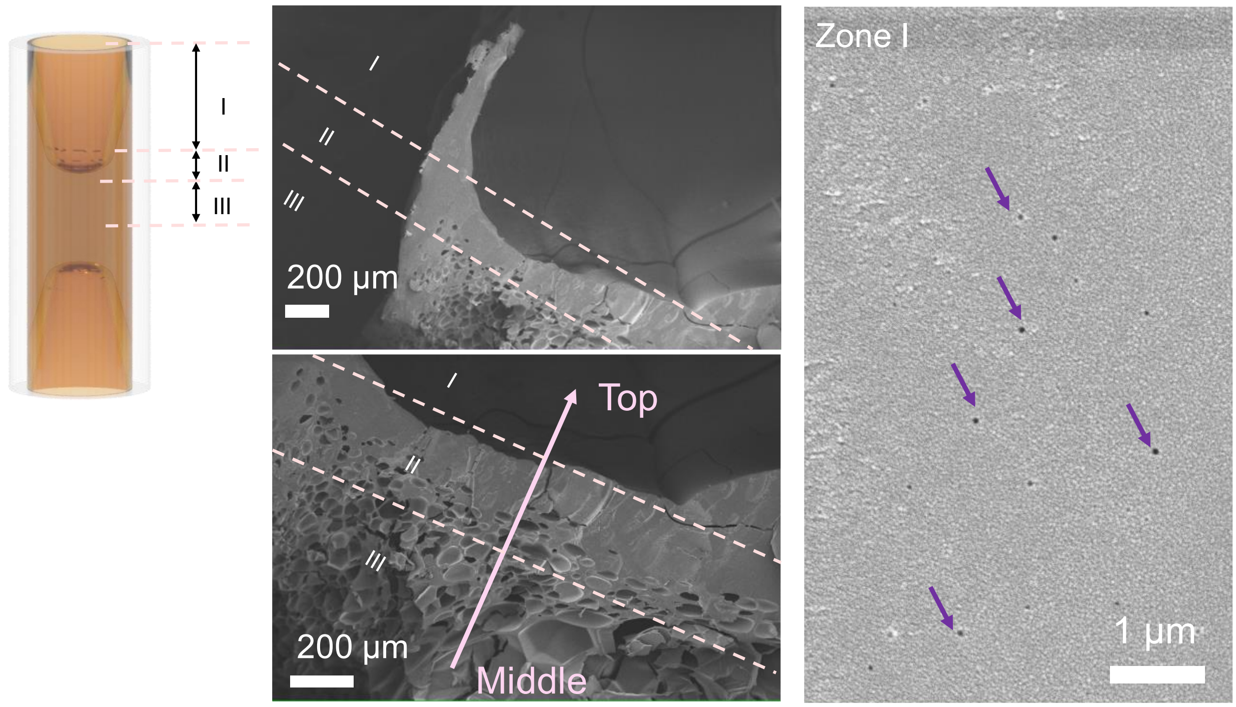

Supplementary Figure 19. The microstructures of hydrogels in the intermediate state during the dehydration.

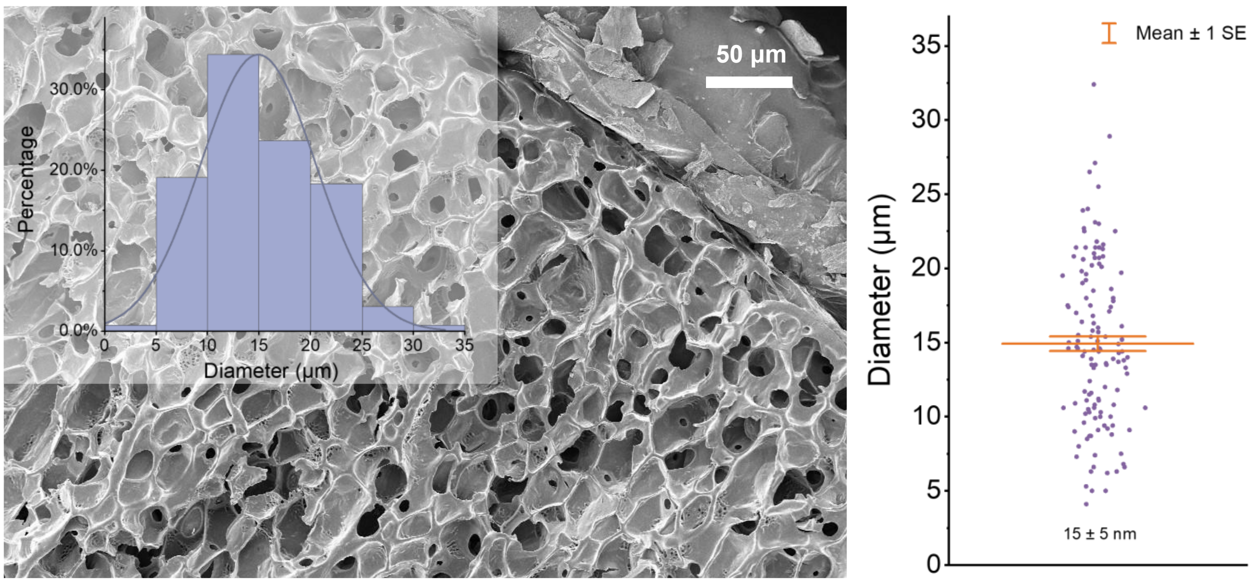

Supplementary Figure 20. The structural features of the lyophilized virgin hydrogels. Pore size distribution is based on statistical analysis of SEM images.

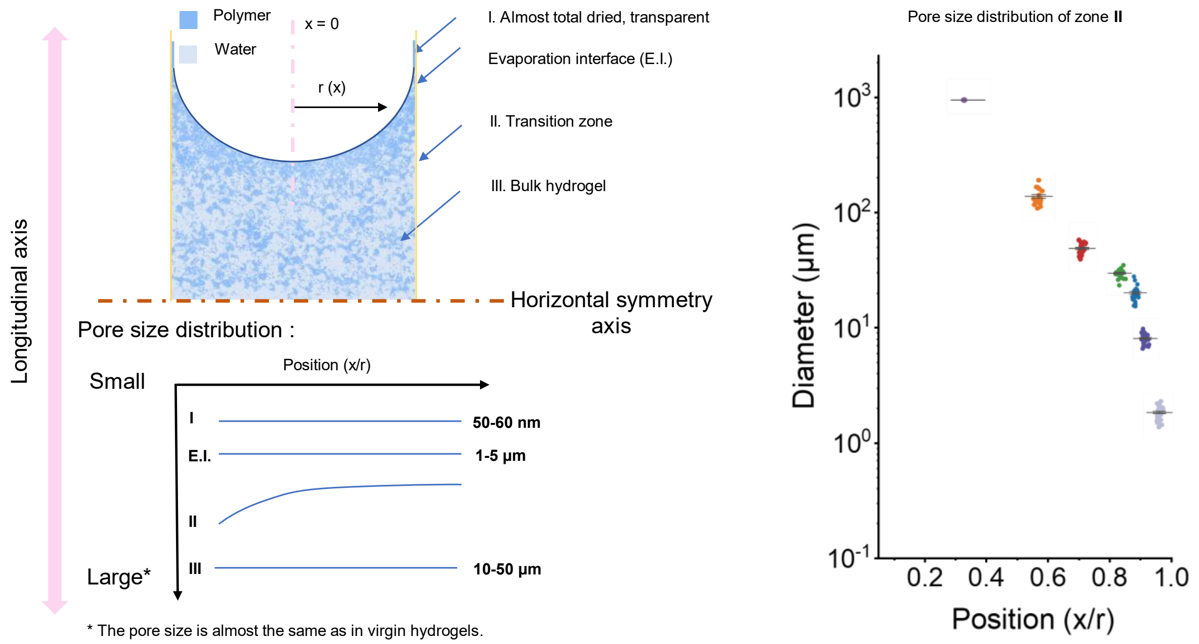

Supplementary Figure 21. An illustration of the microstructures for the hydrogels in the intermediate state during the dehydration. The pore size is determined by the statistical analysis of lyophilized samples.

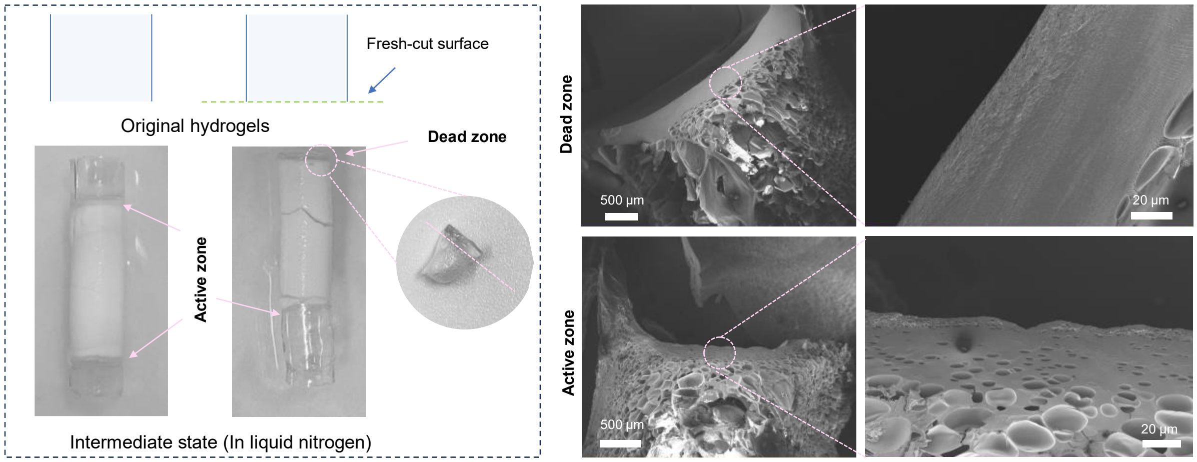

Supplementary Figure 22. The migration of air-hydrogel interface is regulated due to the sensitive to initial characteristics. The images depict hydrogels undergoing dehydration, preserved in liquid nitrogen and subsequently extracted from the models. With the presence of a fresh-cut surface, the air-hydrogel interface still moved inwards from both sides during the initial stage of dehydration. A dense and smooth dead zone was then quickly generated and observed using SEM images around the lyophilized virgin surface. The porous structure within the active zone facilitated a continual migration process of the air-hydrogel interface.

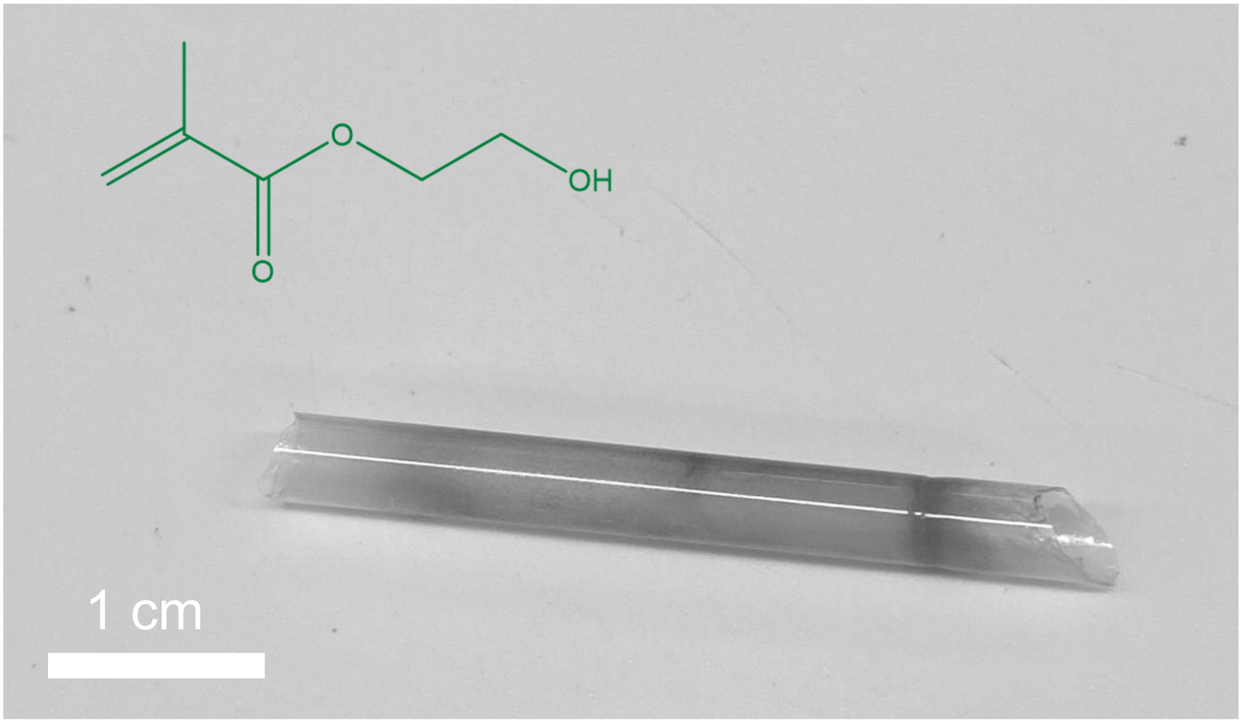

Supplementary Figure 23. A neutral monomer (take 2-hydroxyethyl methacrylate as an example) was applied and similar hollow xerogels were observed.

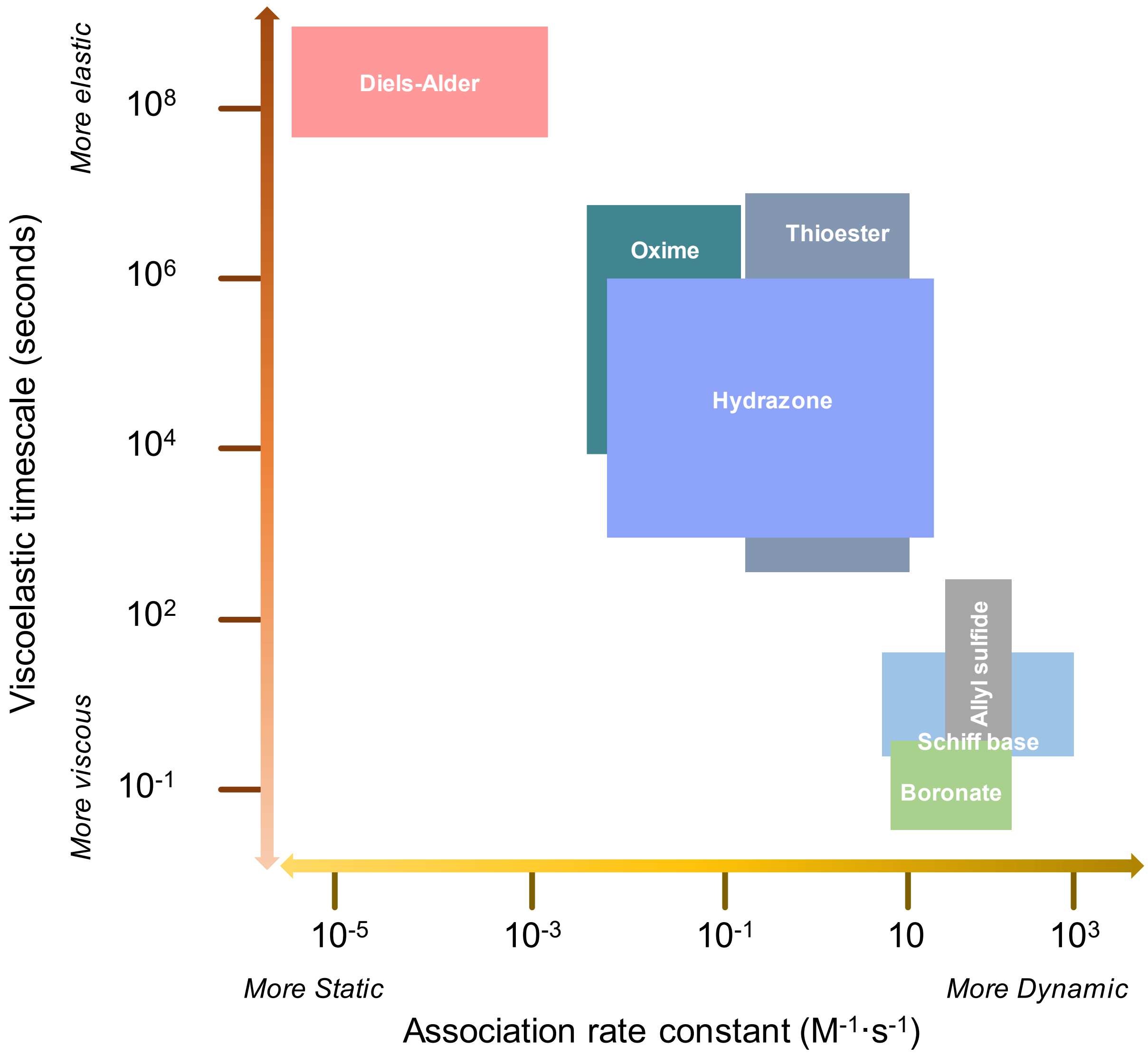

Supplementary Figure 24. A summary of the association rate constants and viscoelastic timescales of a diversity of dynamic bond types[1, 2, 3, 5, 6, 7, 8, 9, 10, 11, 12, 13, 14, 15, 16, 17, 18, 19, 20, 21, 22].

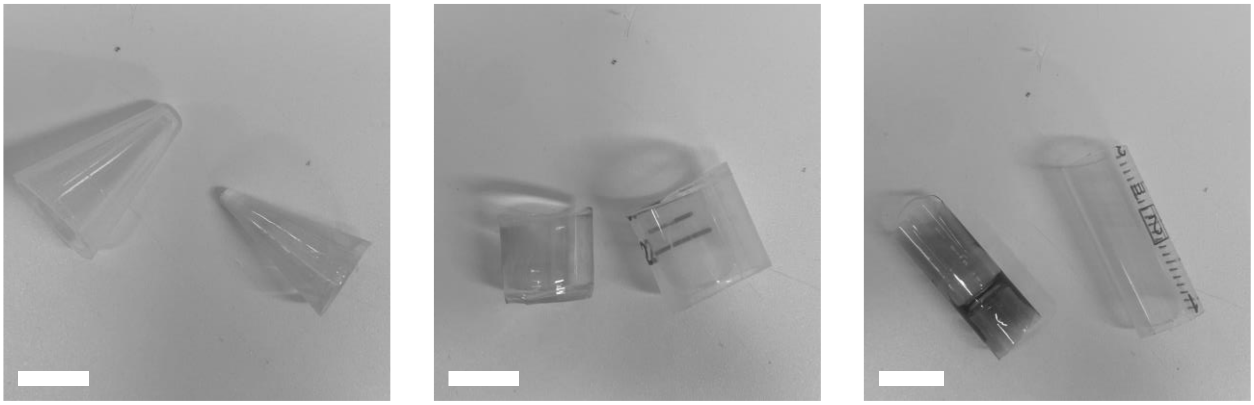

Supplementary Figure 25. The hollow xerogels with different diameters and shapes. Scale bars are 1 cm.

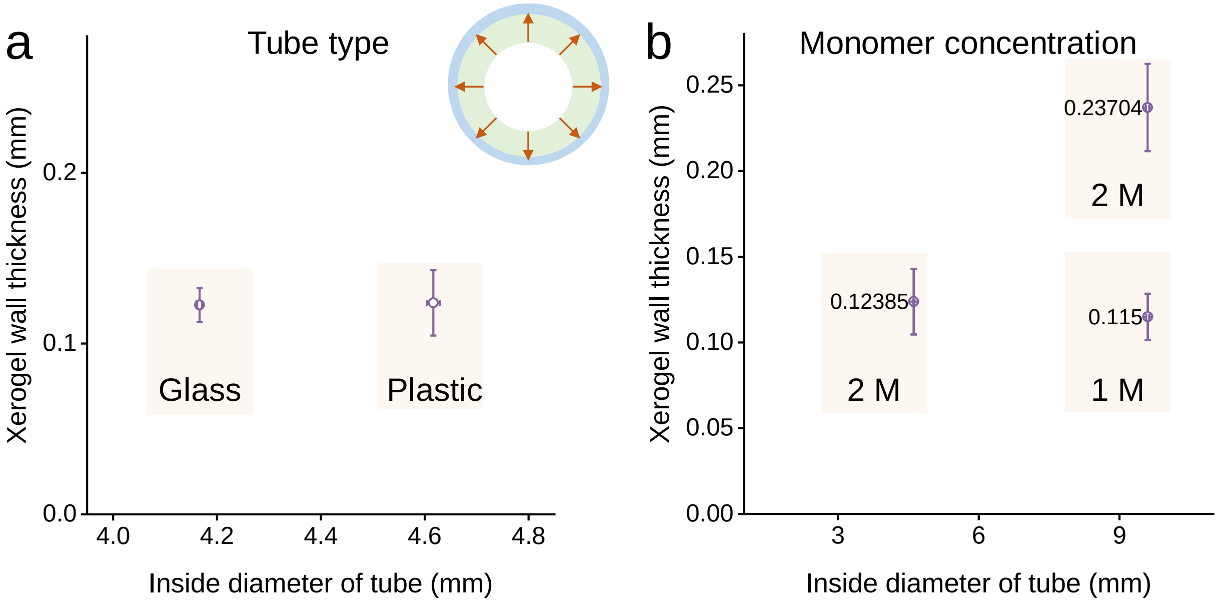

Supplementary Figure 26. The relationship between the xerogel wall thickness and the applied tube. a). Two tubes of different materials with comparable diameters were utilized. The xerogel hollow tubes exhibited a similar thickness. The monomer concentrations were 2 M. b). Two tubes with different diameters were utilized. As xerogel wall generation is contingent upon the accumulation of polymers along the radial direction, it was found that the different xerogel wall thicknesses could be achieved in different systems by adjusting the diameter and polymer fraction. The initial monomer concentrations are labelled. Error bars are SD. Individual sample numbers N = 3.

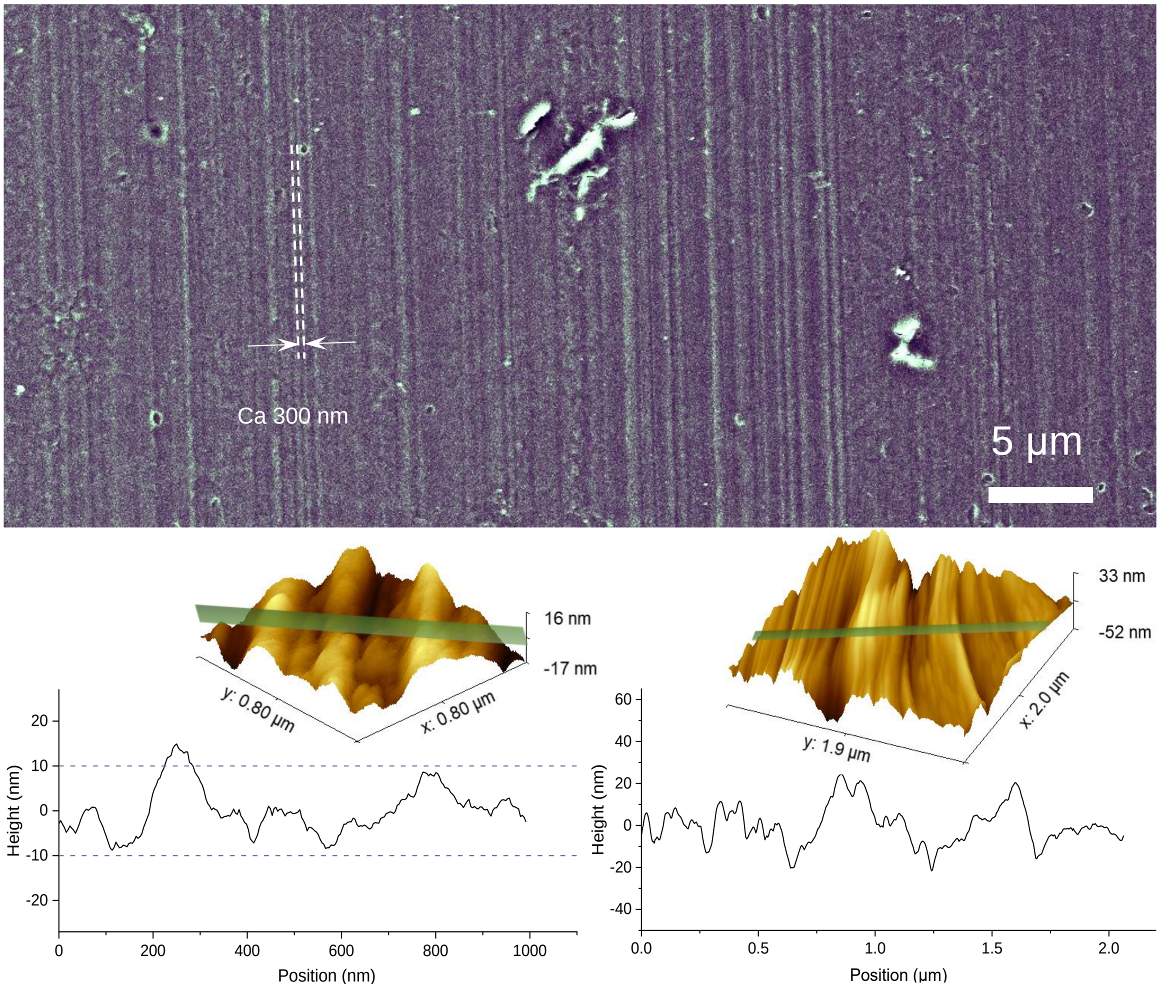

Supplementary Figure 27. The surface morphology of the inner surface of plastic tubes with nanopatterns. The SEM image (false color), AFM image and height information of the inner wall surface with patterns are presented.

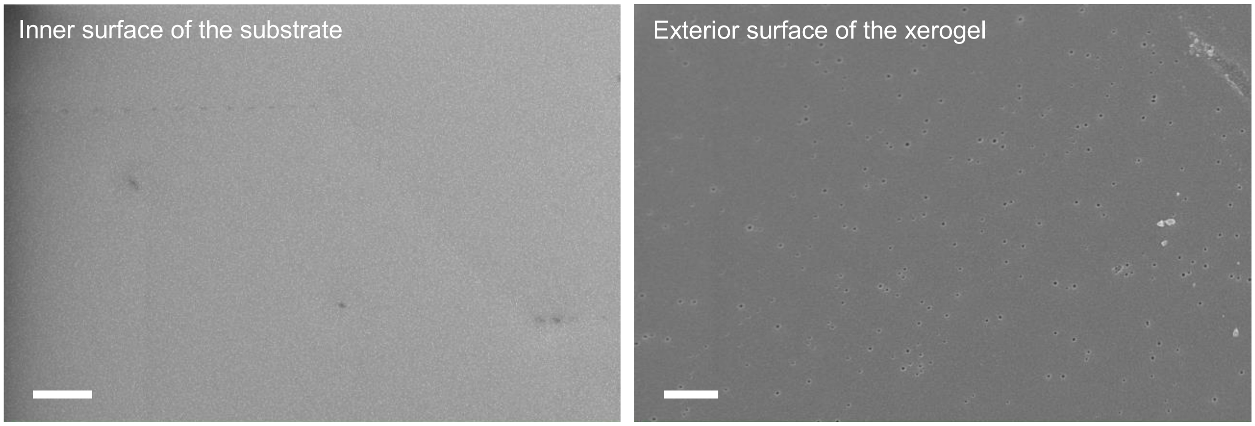

Supplementary Figure 28. The SEM images show the smooth inner wall surface of the substrate and the smooth exterior surface of the resulting hollow xerogel tubes. Scale bars are 1 μm.

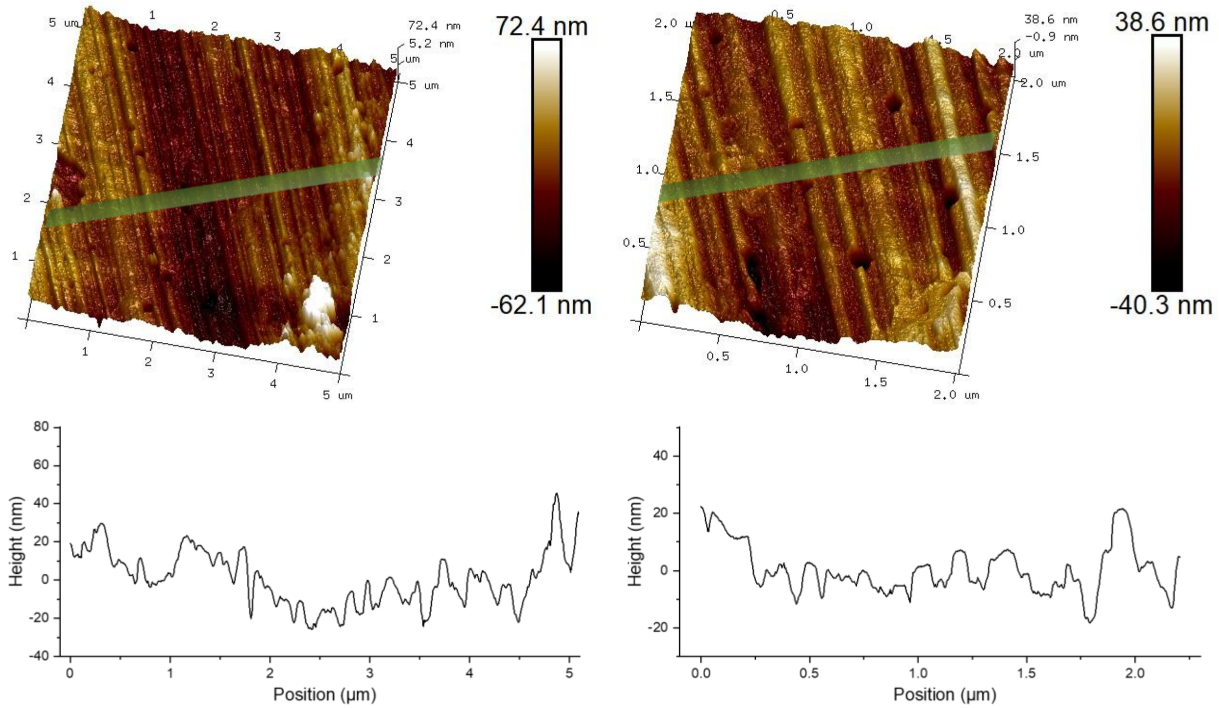

Supplementary Figure 29. The AFM images for the outer surface of hollow xerogel tubes with periodic trenches. The height data was acquired along the green line in the AFM image.

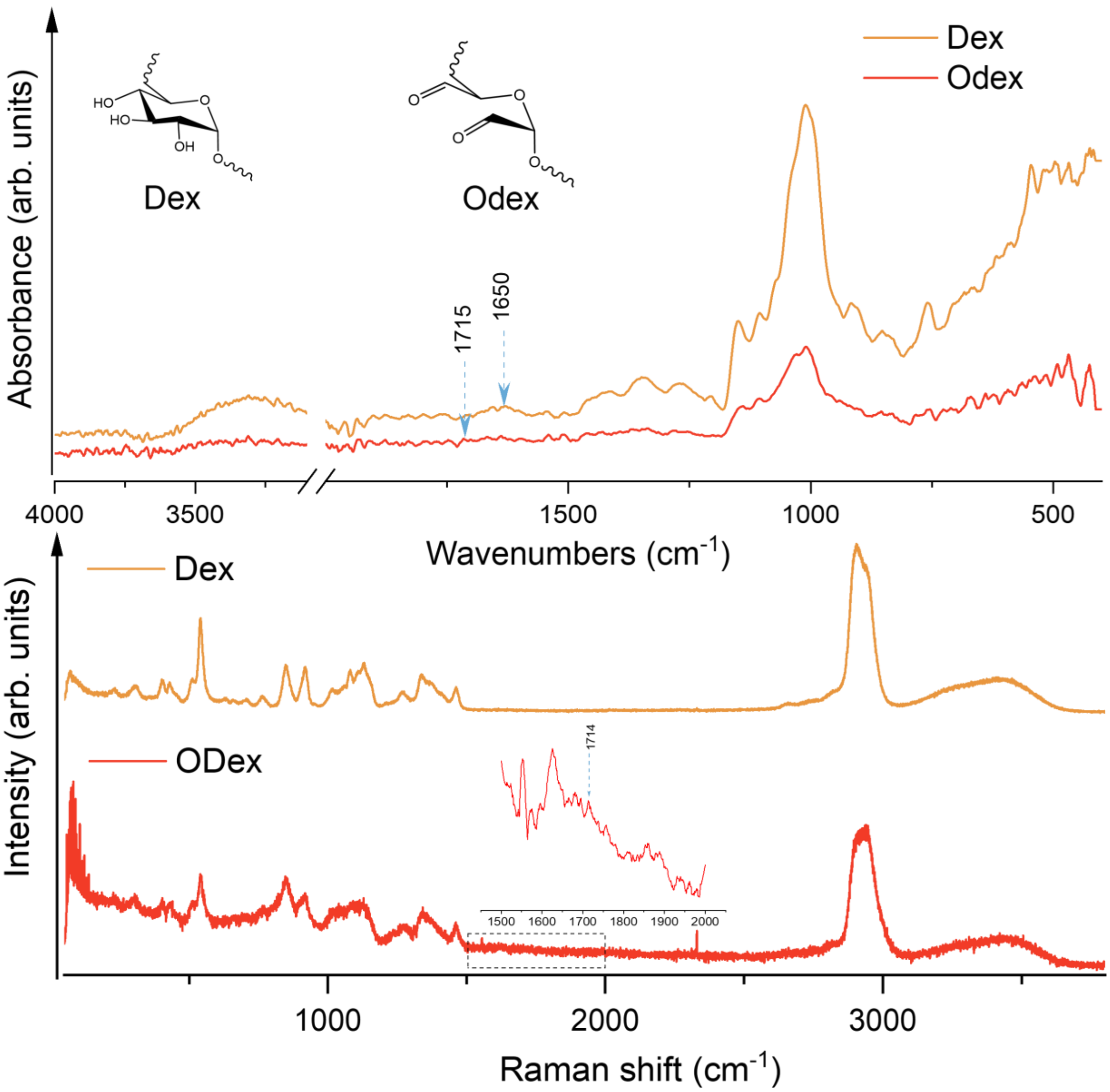

Supplementary Figure 30. The FT-IR spectra and Raman spectra of Dex and Odex samples. The aldehyde bond at 1715 cm^{-1} appeared in the oxidation product, indicating the formation of Odex.

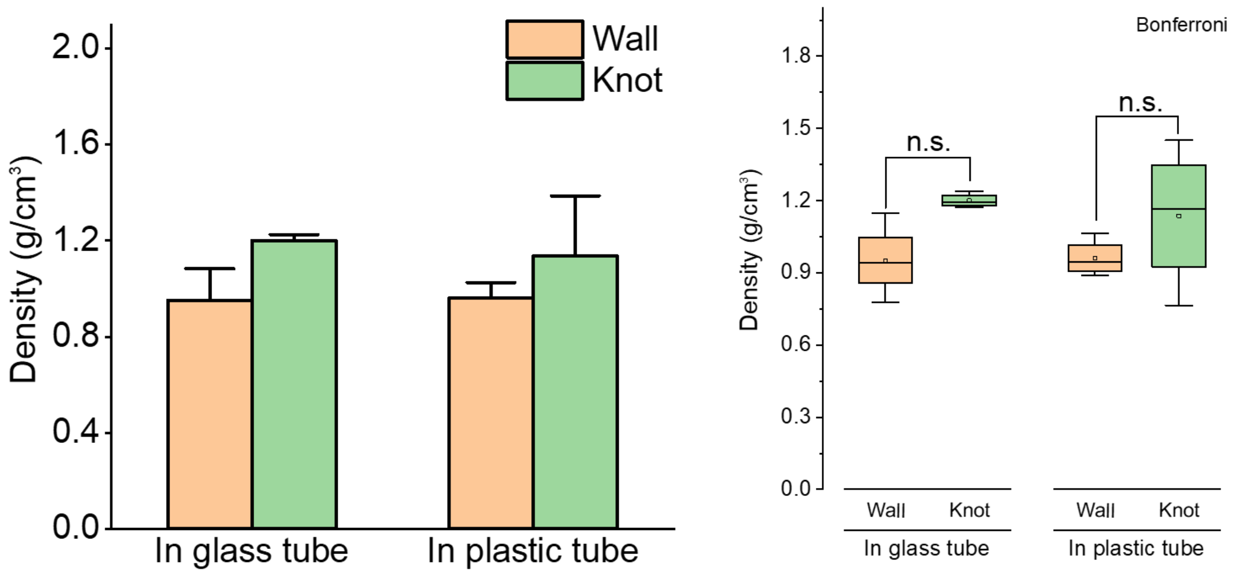

Supplementary Figure 31. The influence of substrates on the resulting xerogel hollow tubes. Error bars are SD. Individual sample numbers N = 5.

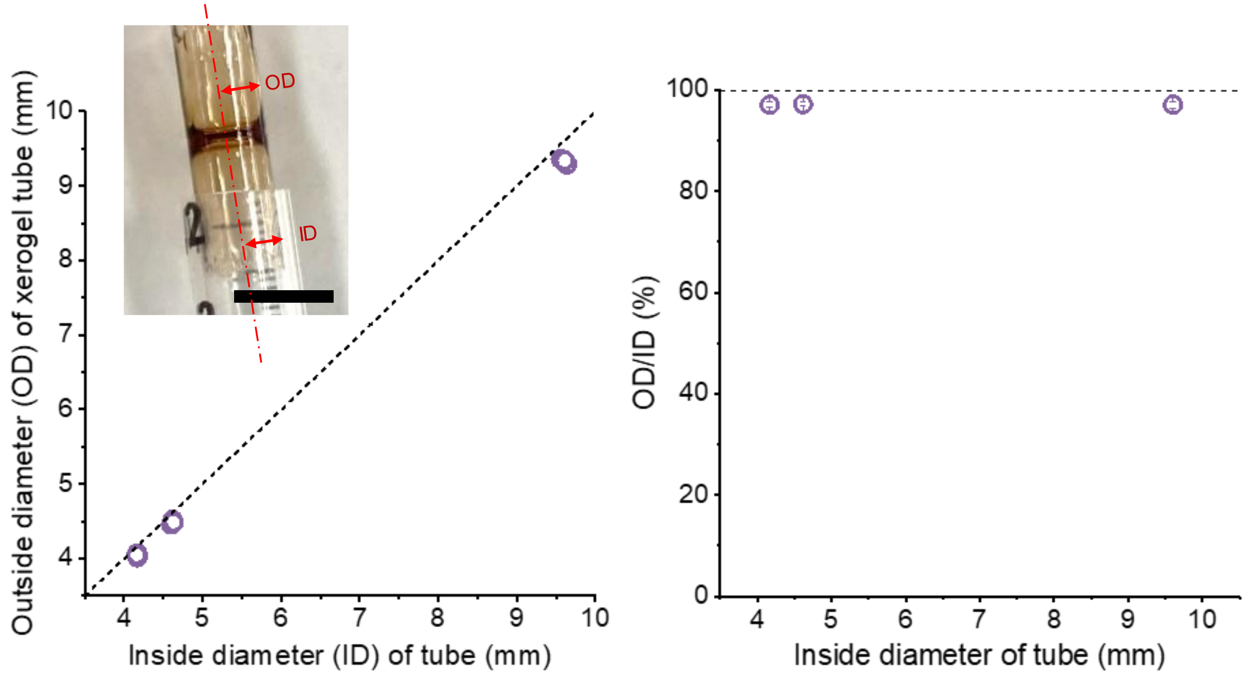

Supplementary Figure 32. The diameters of the tubes and the resulting xerogels. The data were collected using vernier caliper. Due to the deformation induced by measuring force and Abbe error, it can be assumed that the difference between ID and OD is negligible. Scale bar is 5 mm. Error bars are SD. Individual sample numbers N = 3.

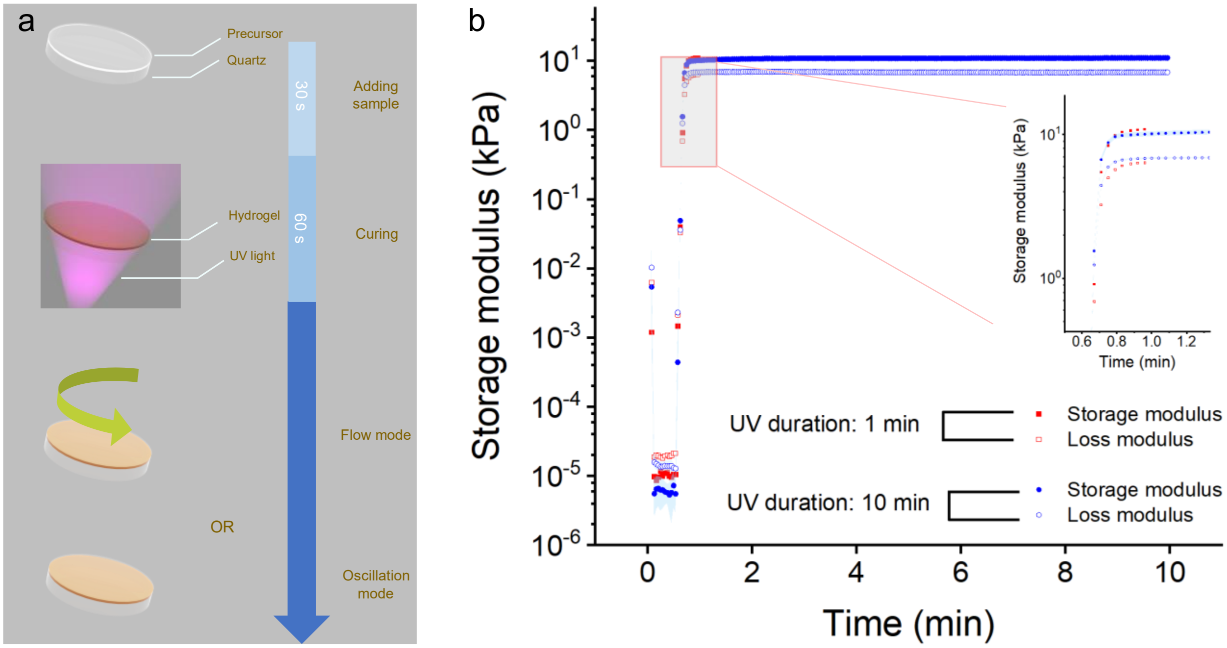

Supplementary Figure 33. An optimized procedure to test the viscosity of hydrogels. a). A standard procedure to in-situ determine the rheological properties of hydrogels. b). The influence of cure time on the rheological properties of hydrogels. It was found that the fast polymerization was almost fully completed within 1 min. Therefore, a short cure time was adopted in our experiments to avoid the influence of hydrogel dehydration in the test.

Supplementary Note 1 - The calculation of cohesion energy

The cohesion energy was calculated to characterize the stability of the hydrogel based on the rheological properties[23, 24, 25]. Generally, the energy can be determined by the critical parameters that represent the transition between the linear viscoelastic regions (LVR) and non-linear viscosity regions. The parameters are critical strain (γ_c), critical shear stress (σ_c) and the storage modulus value of the LVR ($G_c^{'}$). The cohesion energy (E_c) can be related to shear stress:

$$E_c = \int_0^{\gamma_c} \sigma(\gamma)\, d\gamma \tag{1}$$

Critical strain is the deformation limit beyond which the hydrogel response becomes increasingly nonlinear viscoelastic. In the oscillatory test the shear stress is related to the strain by the storage modulus (G'):

$$\sigma = \gamma \cdot G' \tag{2}$$

Taking into account that in LVR the G' remains constant and introducing **Equation. 2** into **Equation.1**, the final integration is given:

$$E_c = \frac{1}{2} G_c' \gamma_c^2 \tag{3}$$

As for the method to determine the critical parameters, a strain sweep is used. For a single frequency and temperature of a stable materials:

$$\sigma = G' \gamma^n \tag{4}$$

where n is the index and when n = 1 is for a line. The stress-strain relationship of a material has no stress with no applied strain, so the intercept, b, is always zero and it is not included. If we take the log of both sides:

$$log(\sigma) = log(G' \gamma^n) \tag{5}$$

Expand the right side using the rules of logarithms:

$$log(\sigma) = nlog(\gamma) + \log(G') \tag{6}$$

Equation. 6 is the stress strain relationship with n = 1 in the linear region. Thus the derivative drops below 1 at the critical strain and the end of the linear region can be very clear in the plot. An example is given in Supplementary Figure 34.

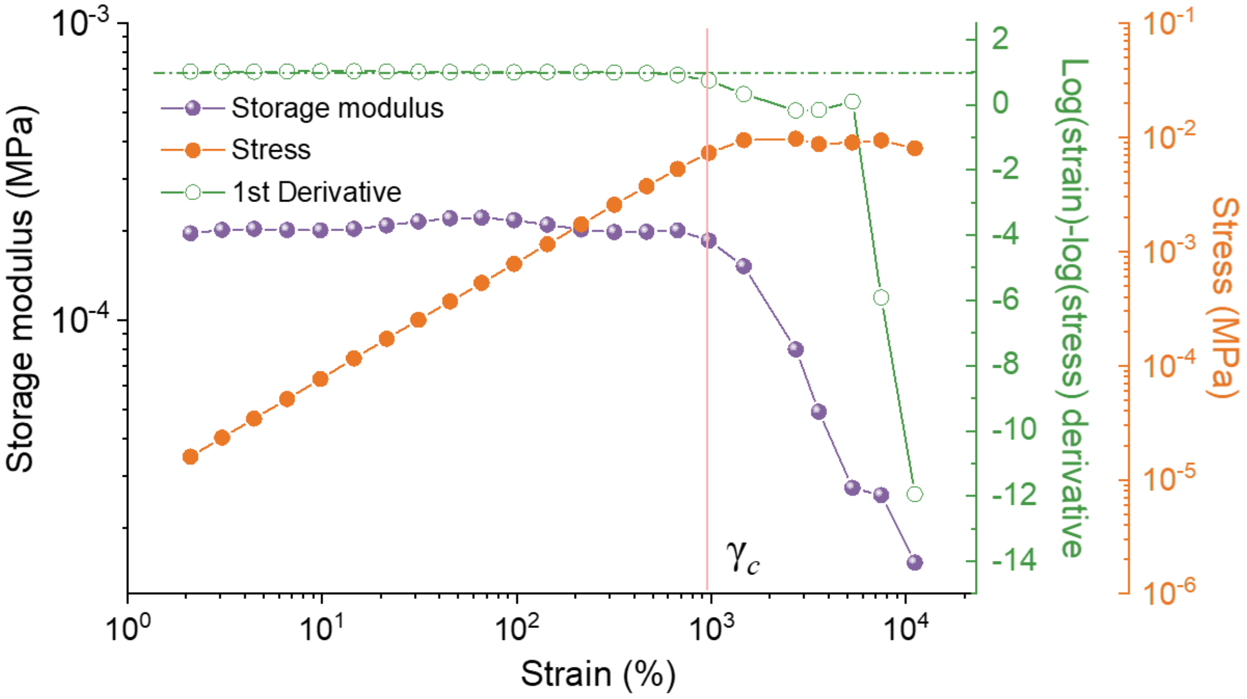

Supplementary Figure 34. A typical strain sweep of hydrogel at 25°C and 0.1 rad/s angular frequency. The stress (orange) and derivative of log stress vs log strain are shown (green). The derivative is close to 1 in the liner region and deviate from 1 at the critical strain. The critical strain marked is about 970% and the derivative is 0.97.

Supplementary Note 2 - The calculation of adhesion energy

The adhesion energy (E_a) is applied to evaluate the interaction between the hydrogel matrix and the inner wall of the tubes. Here, an in-situ method is built as illustrated in Supplementary Figure 35.

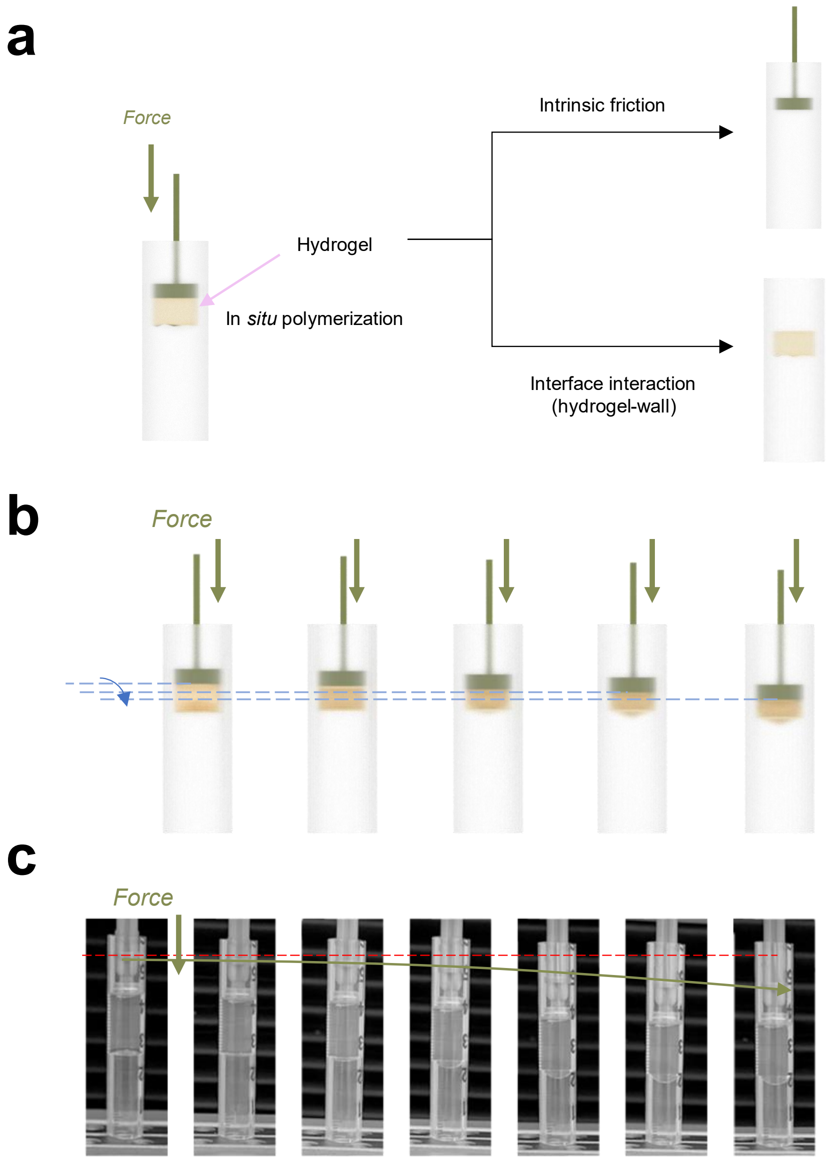

Supplementary Figure 35. A method for determining the interface interaction. a). Illustration of an in-situ method for assessing the interaction strength at the interface. b). Illustration of the slipping around the interface due to applied external force. c). Snapshots during the experiments. The accumulation of force and strain induces a slipping around the interface.

As for a system consisting of the hydrogel and movable head, the overall force (F_{all}) is equal to the sum of the intrinsic friction (F_{fr}) belonging to the movable head and the interface interaction (F_{int}) belonging to the hydrogel-wall interface, expression as below:

$$F_{all} = F_{fr} + F_{int} \tag{7}$$

It should be noted that the moving speed (on the order of 10^{-8}~10^{-10} m/s, Supplementary Figure 37) of hydrogel-air interface is much lower than the lower detection limit of the mechanical testing machine. On the other hand, the peeling force was highly dependent on the peeling force[26, 27, 28]. Herein, we use the working curve and extrapolation method to approximate the values. First, the static friction (F_s) of the moving head is determined (Supplementary Figure 38), while the kinetic friction (F_k) is consider to be independent to the moving speed[29]. The working curve of intrinsic friction is then given by extrapolation method (Supplementary Figure 39, $R^2 > 0.91$). Since there is no slip at the hydrogel-wall interface, the following assumption is reasonable:

$$F_{fr} = F_s \tag{8}$$

Therefore, the intrinsic friction of the system at low speed can be expected.

As for the displacement force curve of the entire system. the F_{all} should be the values of applied force, while here we take the value of static friction of the entire system as F_{all} under the assumption of non-slipping condition (Supplementary Figure 40). The working curve of force versus speed is then given by extrapolation method as well. Herein, we can obtain the value of F_{int} based on **Equation. 7**, while the speed is regarded as the moving speed of hydrogel-air interface. The E_a is defined as:

$$E_a = \frac{F_{int}}{A} \tag{9}$$

where A refers to the contact area of the hydrogel-wall interface (Supplementary Figure 41, $R^2 > 0.95$). The relationship between E_a and the speed of movement has also been established on the basis of the experimental data (Supplementary Figure 42, $R^2 > 0.92$).

For the hydrophilic substrates, which in our experiment refers to the glass, cohesive failure rather than adhesive failure was found (Supplementary Figure 45). Therefore, we believe that the

dynamic hydrogel-wall interface was robust enough and a detailed discussion is not necessary in the current work.

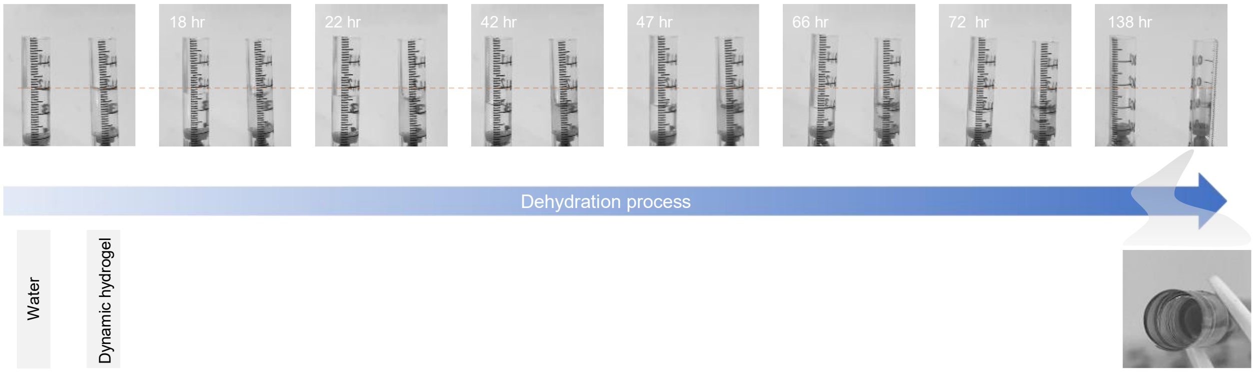

Supplementary Figure 36. A comparison of dehydration process. The left one is pure water while the right one is dynamic hydrogel. A hollow tube xerogel was formed as the result of the dehydration of dynamic hydrogel.

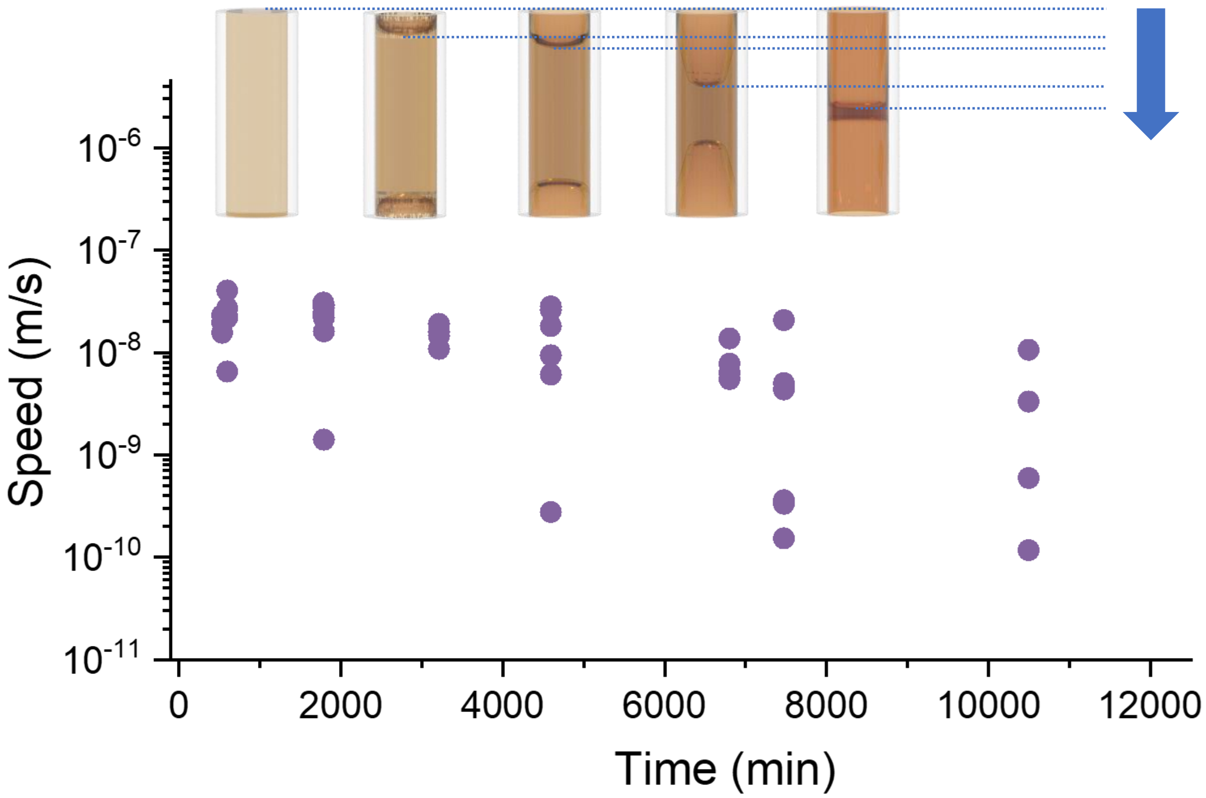

Supplementary Figure 37. The moving speed of hydrogel-air interface during dehydration. The speed refers to the average speed during the certain time periods. The central point at the interface was selected to record the displacement. The dynamic hydrogels with 2 M monomer concentration in a plastic tube with open ends were applied.

The Reynolds number (Re) of this system could be approximately calculated as below

$$Re = \rho v \frac{d}{\mu} \tag{10}$$

Here, the ρ is the density of hydrogels (~1000 kg/m^3), v is the flow speed (set as 10^{-7} m/s), d is the characteristic linear dimension (for plastic tube is the diameter, 4.6×10^{-3} m) and μ is the dynamic viscosity of hydrogels (10^4 Pa·s). Thus the Re in our system is around 4.6×10^{-11}.

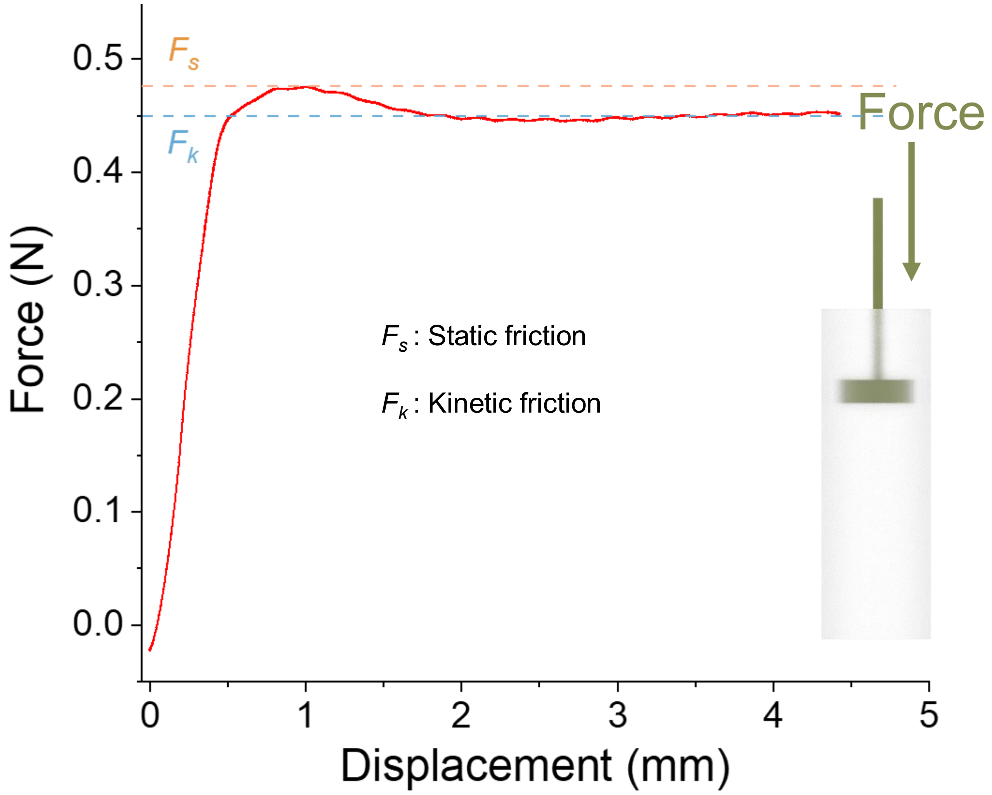

Supplementary Figure 38. A typical displacement-force curve of intrinsic friction.

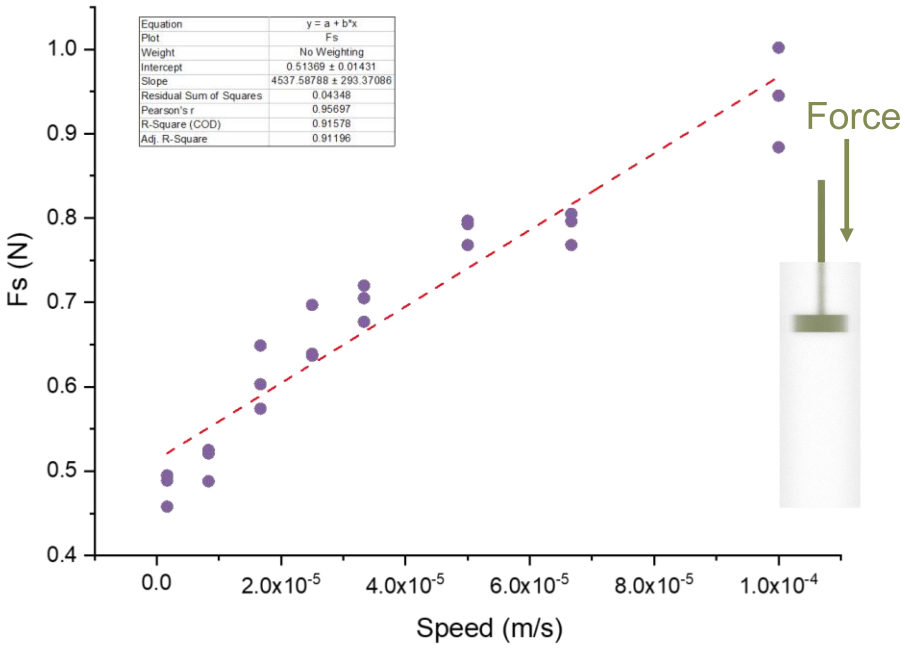

Supplementary Figure 39. The working curves of static friction as the function of speed.

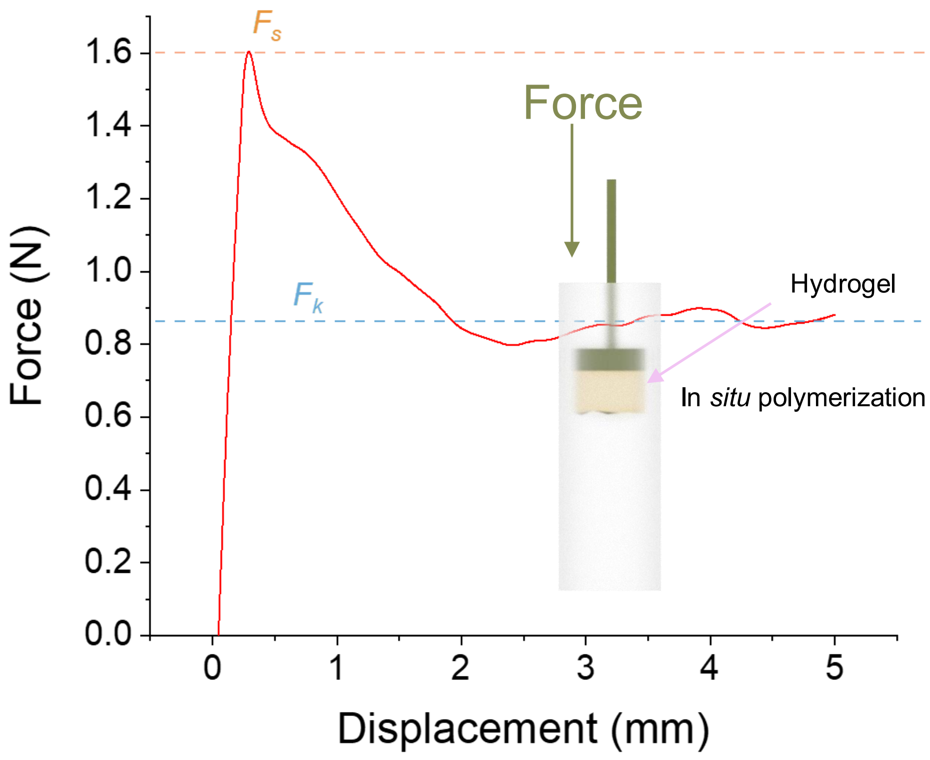

Supplementary Figure 40. A typical displacement-force curve of the hydrogel in tubes.

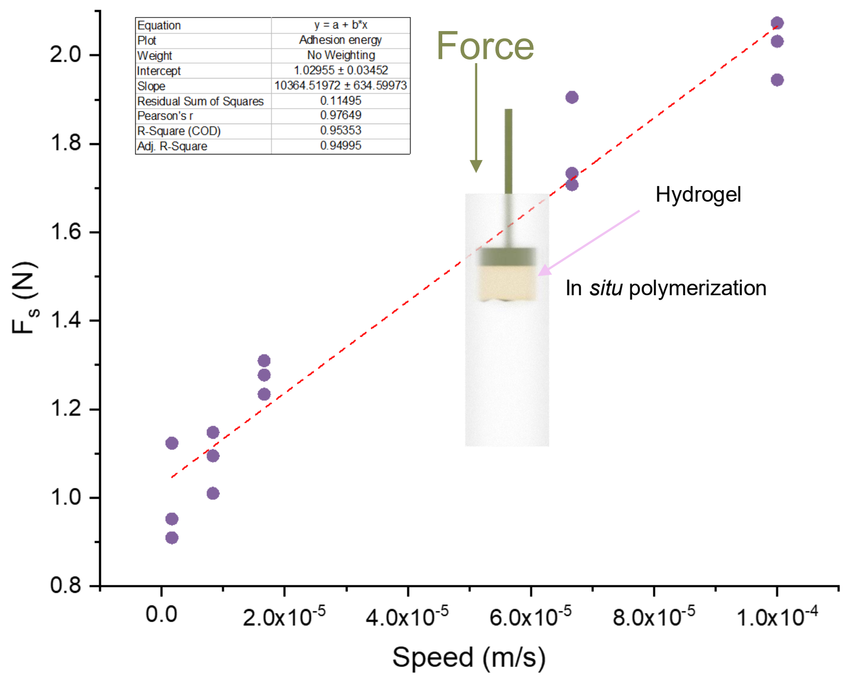

Supplementary Figure 41. The working curves of static friction as the function of speed.

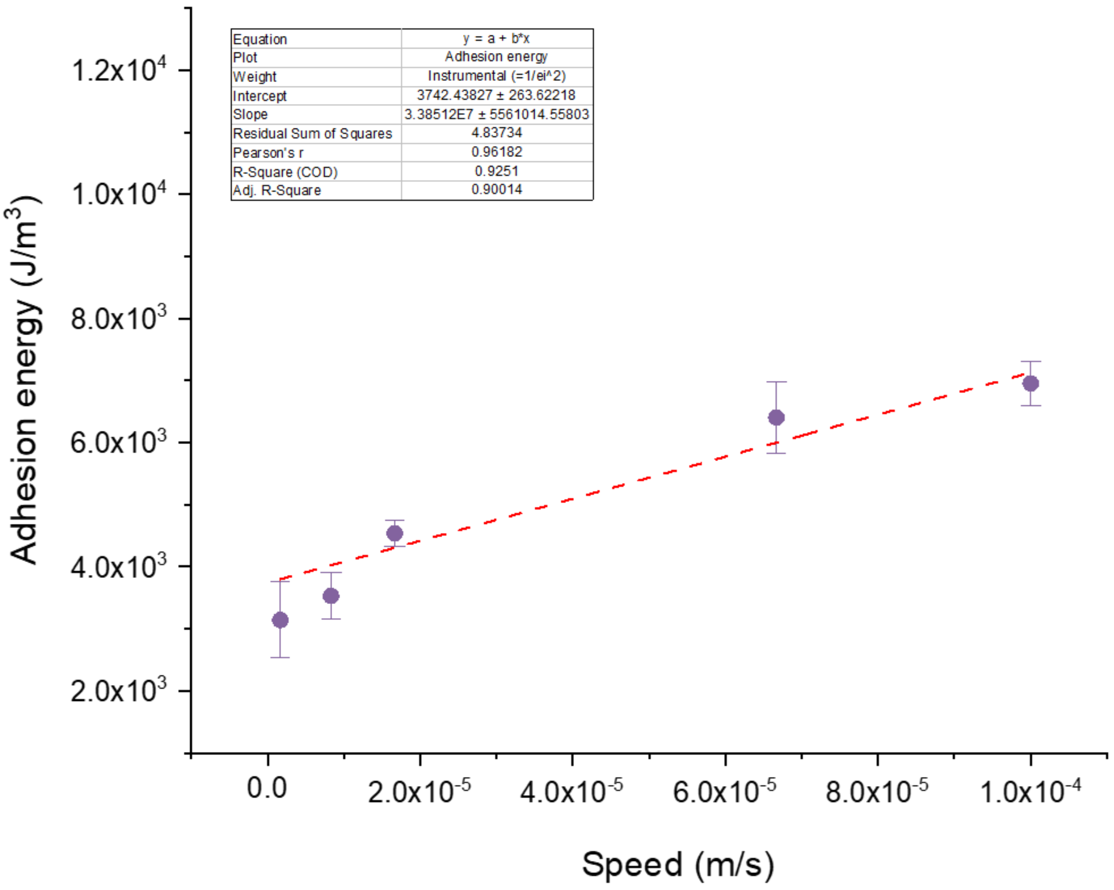

Supplementary Figure 42. The working curves of static friction as the function of speed.

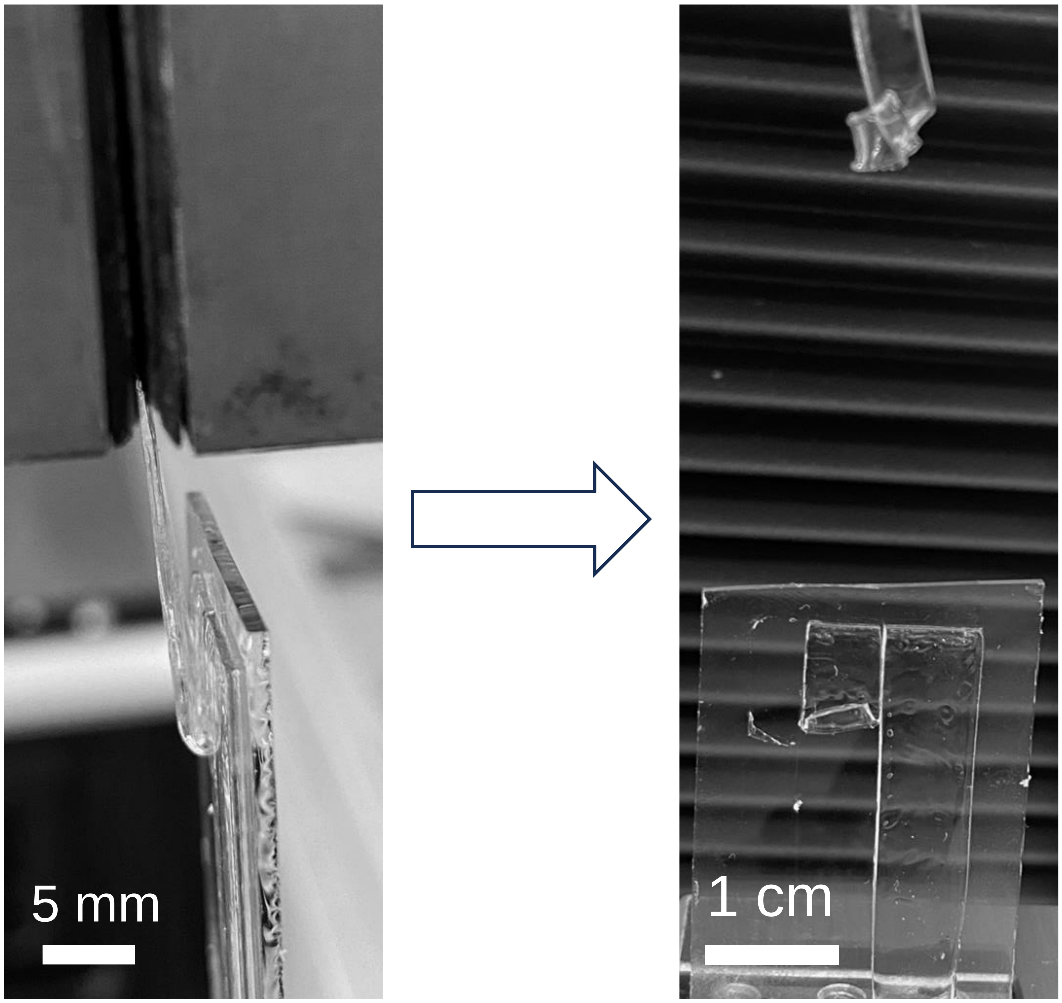

Supplementary Figure 43. The cohesive failure of dynamic hydrogels in 180° peeling test. The moving speed is 0.1 mm/min. Here, we use the slide glass to approach interaction between the dynamic hydrogels and the inner wall of glass tubes. The cohesive failure indicates the adhesion energy exceeds cohesion energy in the system. The moving speed is 0.1 mm/min.

Supplementary Note 3 – Scaling law of adhesion energy *(E_a) and cohesion energy (E_c)*

During dehydration, the water content of the hydrogel evolves over time and thereby both E_a and E_c change simultaneously. First, the polymer fraction (Φ) is defined as the following:

$$\phi = \frac{V_{polymer}}{V_{gel}} = \frac{m_{polymer}/\rho_{polymer}}{m_{gel}/\rho_{gel}} \tag{11}$$

Here, the weight of dry samples was measured and denoted by $m_{polymer}$. The weight of various hydrogel samples was measured and denoted by m_{gel}. The density of drying polymer $\rho_{polymer}$ is taken to be the density of acrylamide, which is 1.13 g/cm^3. The density of gel ρ_{gel} is approximately 1 g/cm^3.

Then, we evaluate the scaling behavior of E_a or E_c as a function of Φ. A power function, as a typical homogeneous function, is used to bridge the scaling relationship between E_a or E_c and Φ, as defined below:

$$E_a or\ E_c = f(\phi) = a\phi^b \tag{12}$$

Combined with the real dehydration behavior or hydrogels in tube, the intercepts of the working curves of E_a or E_c versus speed were used to extrapolate the values at speed = 0 m/s. Thus, regression analysis can be used to assess whether the scaling relationship between E_a or E_c and Φ is obvious.

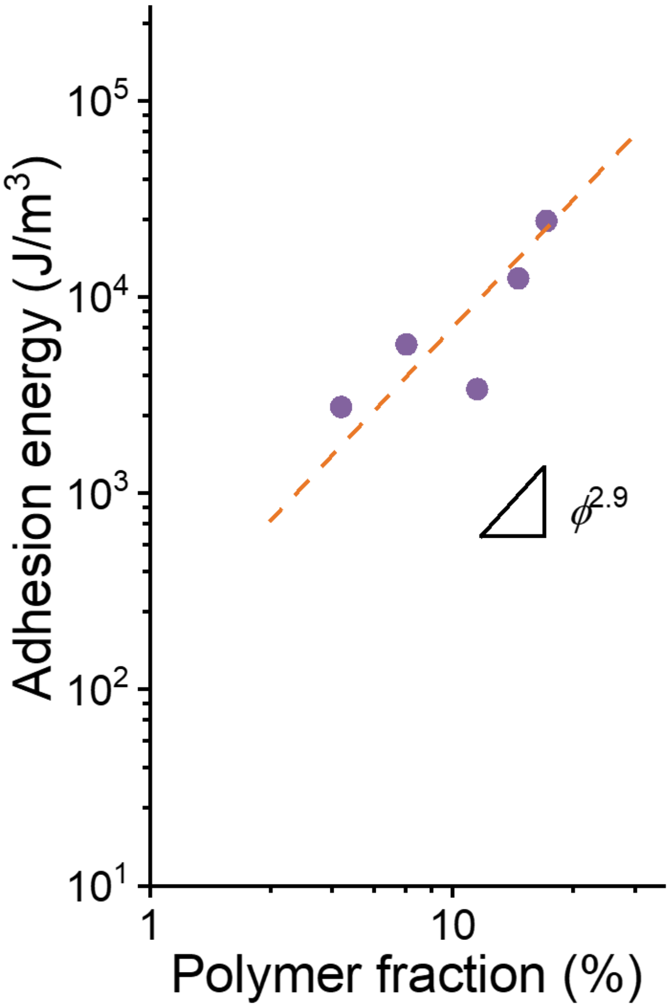

Supplementary Figure 44. The scaling relation between adhesion energies and polymer fraction.

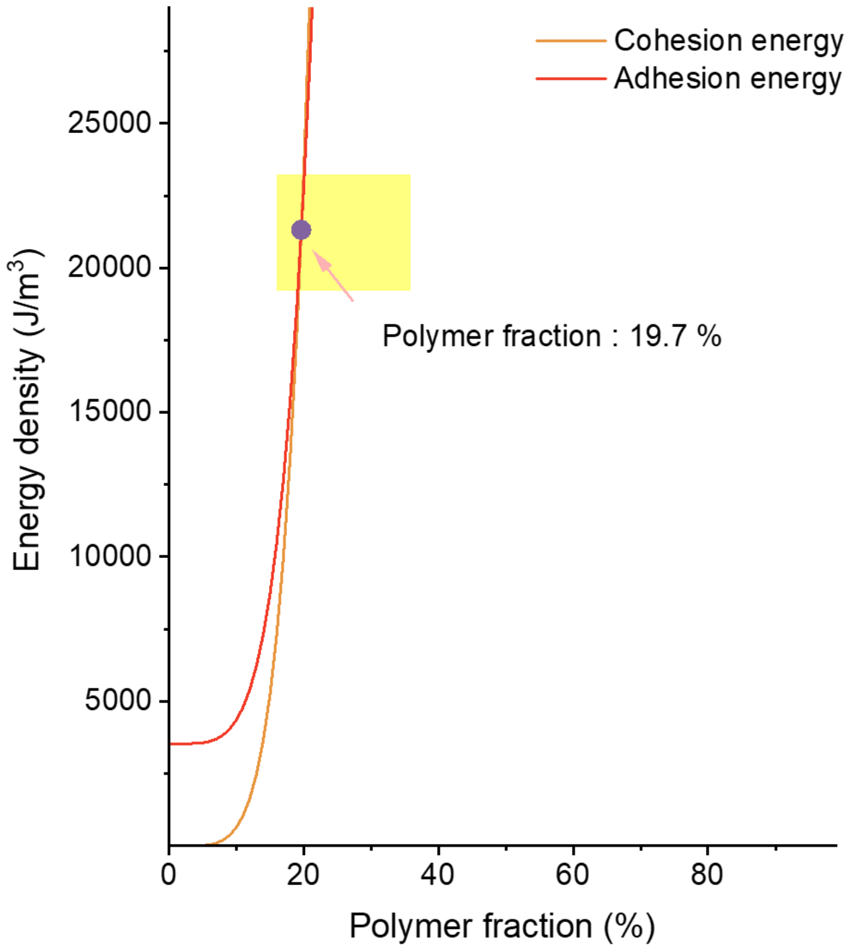

Supplementary Figure 45. The crossover point between cohesion energy and adhesive energy. The mathematical description of the energy density-polymer fraction was given based on the scaling law. The crossover point was approximately 19.7% of the polymer fraction, indicating that the cohesion energy exceeded the adhesion energy at high polymer fractions. This result was in good agreement with the experimental results which showed that for 4 M dynamic hydrogels (polymer fraction 20.4%) the hydrogel-wall interface failed, resulting in isotropic shrinkage. As for the continuity of hydrogel dehydration and deformation under high polymer fraction, reported work has demonstrated a shape increase of E_a under lower water content (high polymer fraction)[30]. This also enhances the stability of the dehydration system.

Supplementary Note 4 - The concentration effect of dynamic bonds concentration during dynamic dehydration of hydrogels

Due to the long period of dehydration, the dynamic bonds based on boronate esters could be oxidized[31]. To clarify this point, we prepared the dynamic hydrogels with different crosslinking densities while keeping the monomer concentrations the same. It was found that the low crosslinking densities (or low boronate ester concentrations) could cause the dynamic hydrogels to flow out of the tubes (Supplementary Figure 46). As there was no obvious flow of dynamic hydrogels in the experiments, the influence of the oxidation induced concentration effect of boronate esters was not emphasized. In other words, we can estimate that the evolution of the dynamic binding concentration was limited.

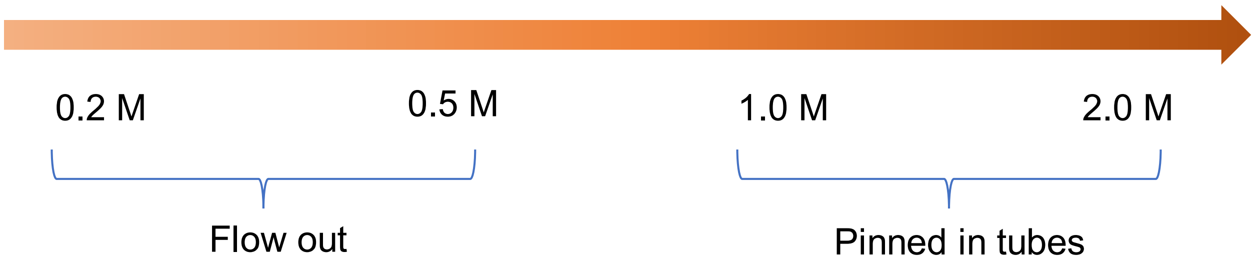

Supplementary Figure 46. The concentration effect of dynamic bonds concentration during dynamic dehydration of hydrogels.

Supplementary Note 5 – Simulation of hydrogel dehydration process in tubes via COMSOL based on a Stefan flow

Vapor molecules emitted from the hydrogel surface diffuse into the air, which is called as vapor diffusion as seen in Supplementary Figure 47. Similarly, air molecules diffuse towards the hydrogel surface due to the concentration gradient, a process known as air diffusion (Supplementary Figure 47). During evaporation, air molecules transport downwards due to the presence of a concentration gradient. As the hydrogel surface-vapor interface is impermeable to air molecules, an upward Stefan flow forms to maintain the vapor pressure at the interface. The convection of air enhanced the dehydration process.

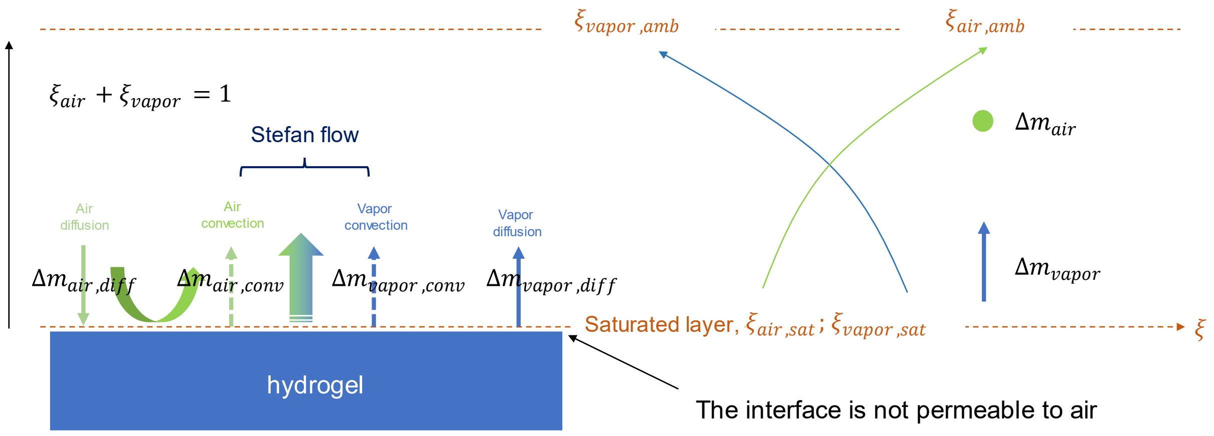

Supplementary Figure 47. Interfacial mass transport mechanisms during hydrogel dehydration near hydrogel surface.

First, the diffusion flow of air must be balanced by a convective flow in opposite direction:

$$\Delta m_{air,diff} = -\Delta m_{air,conv} \tag{13}$$

Where Δm is the local mass flux at the surface due to the phase change.

Considering mass flow in steady state:

$$\Delta m_{air} = \Delta m_{air,diff} + \Delta m_{air,conv} = q_{air} + \rho v \xi_{air} \tag{14}$$

$$\Delta m_{vapor} = \Delta m_{vapor,diff} + \Delta m_{vapor,conv} = q_{vapor} + \rho v \xi_{vapor} \tag{15}$$

Where q is the local mass flux induced by diffusion.

Equation. 13 and **Equation. 14** can be added together to yield

$$\Delta m_{air} + \Delta m_{vapor} = \left(q_{air} + q_{vapor}\right) + \rho v\left(\xi_{air} + \xi_{vapor}\right) \tag{16}$$

Here,

$$\xi_{air} + \xi_{vapor} = 1 \tag{17}$$

Where ξ is the mass fraction of species.

Taking account of the steady state,

$$q_{air} + q_{vapor} = 0 \tag{18}$$

Substituting **Equation. 17**, **Equation. 18** into **Equation. 16**,

$$\Delta m_{vapor} = \rho v = \Delta m_{total} = \Delta m \tag{19}$$

Here, v is the Stefan velocity that we discussed.

In COMSOL, the evaporation flux is defined as,

$$\Delta m = \begin{cases} M_v K(c_v - c_{sat}), & if\, c_v > c_{sat}\; or\; c_l > 0 \\ 0\; otherwise \end{cases} \tag{20}$$

Where M_v is the molar mass of water vapor, K is the evaporation rate factor, c_{sat} is the saturation concentration of the vapor, c_v is the vapor concentration and c_l is the liquid water concentration on surface.

The liquid water concentration on surface is computed by solving the following equation,

$$M_v \frac{\partial c_l}{\partial t} = -\Delta m \tag{21}$$

with the initial condition:

$$c_l(0) = c_{l,int} \tag{22}$$

The latent heat source J_{evap} is obtained by multiplying the evaporation flux by the latent heat of evaporation L_v:

$$J_{evap} = L_v \Delta m \tag{23}$$

Due to the endothermic nature of the dehydration process and the relatively higher thermal conductivity of the tube wall, a gradient Stefan velocity distribution along the radial direction is reasonably observed.

The boundary conditions are illustrated in Supplementary Figure 48. Generally,

$$u = 0, v = v_0, T = T_{amb}\ at\ y = 0 \tag{24}$$

$$u = 0, T = T_{amb}\ at\ y \to \infty \tag{25}$$

where *u* and *v* are the flow velocity in x- and y- direction, respectively. *T* is the temperature.

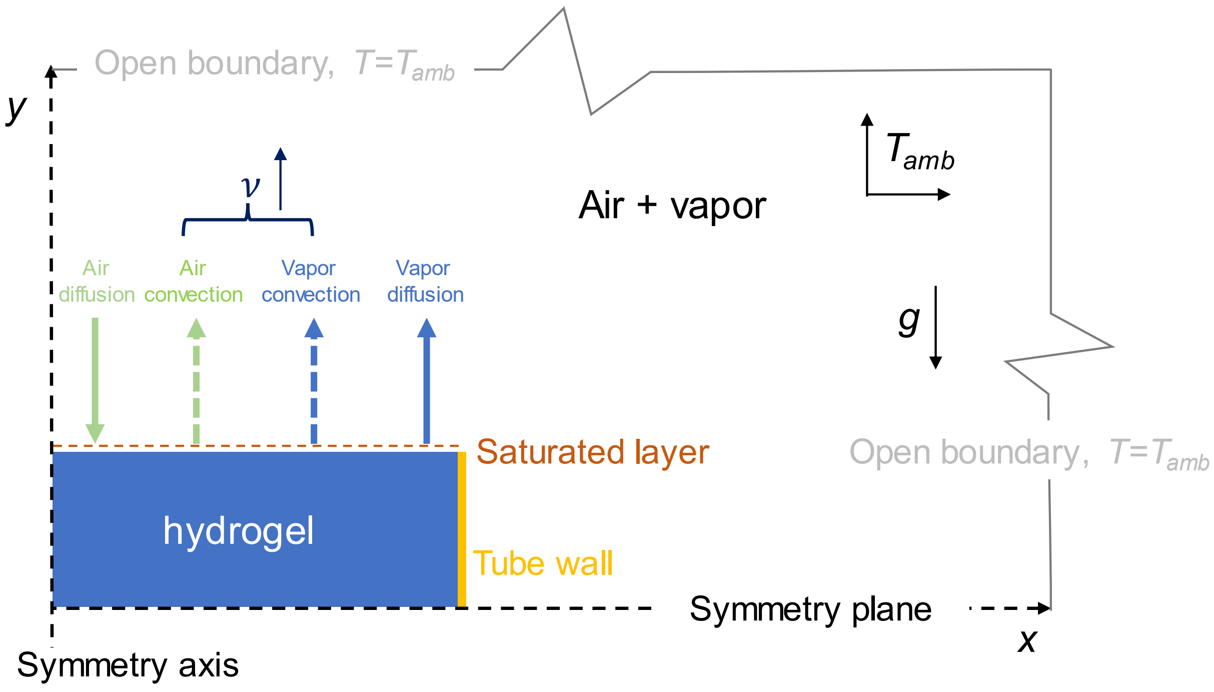

Supplementary Figure 48. Problem domain and boundary conditions.

The initial relative humidity of the environment is assumed to be 20%. A simple approach is to assume that the interfacial temperatures are the same in both phases. The ambient temperature is set at 273.15 K.

Supplementary references

1. Tang S, Ma H, Tu HC, Wang HR, Lin PC, Anseth KS. Adaptable fast relaxing boronate-based hydrogels for probing cell–matrix interactions. *Adv Sci* **5**, 1800638 (2018).

2. van Hurne S, Kisters M, Smulders MM. Covalent adaptable networks using boronate linkages by incorporating TetraAzaADamantanes. *Front Chem* **11**, 1148629 (2023).

3. Kang B, Kalow JA. Internal and external catalysis in boronic ester networks. *ACS Macro Lett* **11**, 394-401 (2022).

4. Paterson SM, Casadio YS, Brown DH, Shaw JA, Chirila TV, Baker MV. Laser scanning confocal microscopy versus scanning electron microscopy for characterization of polymer morphology: Sample preparation drastically distorts morphologies of poly(2-hydroxyethyl methacrylate)-based hydrogels. *J Appl Polym Sci* **127**, 4296-4304 (2013).

5. Akine S, Kusama D, Takatsuki Y, Nabeshima T. Synthesis of tetrafunctionalized pentiptycenequinones for construction of cyclic dimers with a cylindrical shape by boronate ester formation. *Tetrahedron Lett* **56**, 4880-4884 (2015).

6. Collins BE, Metola P, Anslyn EV. On the rate of boronate ester formation in ortho-aminomethyl-functionalised phenyl boronic acids. *Supramol Chem* **25**, 79-86 (2013).

7. Mai V-D, Shin S-R, Lee D-S, Kang I. Thermal healing, reshaping and ecofriendly recycling of epoxy resin crosslinked with Schiff base of vanillin and hexane-1, 6-diamine. *Polymers* **11**, 293 (2019).

8. Jiang L, Tian Y, Wang X, Zhang J, Cheng J, Gao F. A fully bio-based Schiff base vitrimer with self-healing ability at room temperature. *Polym Chem* **14**, 862-871 (2023).

9. Ivanov A, Larsson H, Galaev IY, Mattiasson B. Synthesis of boronate-containing copolymers of N, N-dimethylacrylamide, their interaction with poly (vinyl alcohol) and rheological behaviour of the gels. *Polymer* **45**, 2495-2505 (2004).

10. Engelsma SB*, et al.* Acylazetine as a Dienophile in Bioorthogonal Inverse Electron-Demand Diels–Alder Ligation. *Org Lett* **16**, 2744-2747 (2014).

11. Marozas IA, Cooper-White JJ, Anseth KS. Photo-induced viscoelasticity in cytocompatible hydrogel substrates. *New J Phys* **21**, 045004 (2019).

12. Lou J, Mooney DJ. Chemical strategies to engineer hydrogels for cell culture. *Nat Rev Chem* **6**, 726-744 (2022).

13. Thys M, Brancart J, Van Assche G, Vendamme R, Van den Brande N. Reversible Lignin-Containing Networks Using Diels–Alder Chemistry. *Macromolecules* **54**, 9750-9760 (2021).

14. Safaei A, Terryn S, Vanderborght B, Van Assche G, Brancart J. Toughening and Stiffening in Thermoreversible Diels–Alder Polymer Network Blends. *Macromolecules* ***56*,** (2023).

15. Bongiardina NJ, Long KF, Podgórski M, Bowman CN. Substituted thiols in dynamic thiol–thioester reactions. *Macromolecules* **54**, 8341-8351 (2021).

16. HARANO K, OHIZUMI N, MISAKA K, YAMASHIRO S, HiSANO T. Stereoselective formation of allylic sulfides via two sequential [3, 3]-sigmatropic rearrangements of allylic xanthates and its mechanistic aspects. *Chem Pharm Bull* **38**, 619-624 (1990).

17. Zhang S, Huang D, Lin H, Xiao Y, Zhang X. Cellulose nanocrystal reinforced collagen-based nanocomposite hydrogel with self-healing and stress-relaxation properties for cell delivery. *Biomacromolecules* **21**, 2400-2408 (2020).

18. Sánchez-Morán H, Ahmadi A, Vogler B, Roh K-H. Oxime cross-linked alginate hydrogels with tunable stress relaxation. *Biomacromolecules* **20**, 4419-4429 (2019).

19. St. Amant AH*, et al.* Tuning the Diels–Alder reaction for bioconjugation to maleimide drug-linkers. *Bioconjugate Chem* **29**, 2406-2414 (2018).

20. Carberry BJ, Rao VV, Anseth KS. Phototunable viscoelasticity in hydrogels through thioester exchange. *Ann Biomed Eng* **48**, 2053-2063 (2020).

21. Morgan FL, Fernández-Pérez J, Moroni L, Baker MB. Tuning Hydrogels by Mixing Dynamic Cross-Linkers: Enabling Cell-Instructive Hydrogels and Advanced Bioinks. *Adv Healthcare Mater* **11**, 2101576 (2022).

22. Richardson BM, Wilcox DG, Randolph MA, Anseth KS. Hydrazone covalent adaptable networks modulate extracellular matrix deposition for cartilage tissue engineering. *Acta Biomater* **83**, 71-82 (2019).

23. Alazzawi MK, Rohn CL, Beyoglu B, Haber RA. Rheological assessment of cohesive energy density of highly concentrated stereolithography suspensions. *Ceram Int* **46**, 8473-8477 (2020).

24. Jia Y-G, Zhu X. Self-healing supramolecular hydrogel made of polymers bearing cholic acid and β-cyclodextrin pendants. *Chem Mater* **27**, 387-393 (2015).

25. Williams DJ, Williams PR. Rheology of concentrated cohesive sediments. *J Coastal Res*, 165-173 (1989).

26. Yamada M, Takahashi K, Fujimura N, Nakamura T. Generalized characteristics of peel tests independent of peel angle and tape thickness. *Eng Fract Mech* **271**, 108653 (2022).

27. Gay C, Leibler L. On Stickiness. *Phys Today* **52**, 48-52 (1999).

28. Peng Z, Wang C, Chen L, Chen S. Peeling behavior of a viscoelastic thin-film on a rigid substrate. *Int J Solids Struct* **51**, 4596-4603 (2014).

29. Zhang Q, Qi Y, Hector Jr LG, Cagin T, Goddard III WA. Atomic simulations of kinetic friction and its velocity dependence at Al/Al and α− Al 2 O 3/α− Al 2 O 3 interfaces. *Phys Rev B: Condens Matter* **72**, 045406 (2005).

30. Zhou Z, Lei J, Liu Z. Effect of water content on physical adhesion of polyacrylamide hydrogels. *Polymer* **246**, 124730 (2022).

31. Mirviss SB. Mechanism of the oxidation of trialkylboranes. *J. Org. Chem.* **32**, 1713-1717 (1967).
